# Natural SNP Variation in *GbOSM1* Promotor Enhances Verticillium Wilt Resistance in Cotton

**DOI:** 10.1002/advs.202406522

**Published:** 2024-10-16

**Authors:** Guilin Wang, Dayong Zhang, Haitang Wang, Jinmin Kong, Zhiguo Chen, Chaofeng Ruan, Chaoyang Deng, Qihang Zheng, Zhan Guo, Hanqiao Liu, Weixi Li, Xinyu Wang, Wangzhen Guo

**Affiliations:** ^1^ State Key Laboratory of Crop Genetics & Germplasm Enhancement and Utilization Nanjing Agricultural University Nanjing 210095 China; ^2^ Engineering Research Center of Ministry of Education for Cotton Germplasm Enhancement and Application Nanjing Agricultural University Nanjing 210095 China; ^3^ College of Life Sciences Nanjing Agricultural University Nanjing 210095 China

**Keywords:** cotton, haplotype, NFYA5, osmotin, verticillium dahliae

## Abstract

Osmotin is classified as the pathogenesis‐related protein 5 group. However, its molecular mechanism involved in plant disease resistance remains largely unknown. Here, a Verticillium wilt (VW) resistance‐related osmotin gene is identified in *Gossypium barbadense* (*Gb*), *GbOSM1*. *GbOSM1* is preferentially expressed in the roots of disease‐resistant *G. barbadense* acc. Hai7124 and highly induced by *Verticillium dahliae* (*Vd*). Silencing *GbOSM1* reduces the VW resistance of Hai7124, while overexpression of *GbOSM1* in disease‐susceptible *G. hirsutum* improves tolerance. GbOSM1 predominantly localizes in tonoplasts, while it relocates to the apoplast upon exposure to osmotic stress or *Vd* infection. GbOSM1 confers VW resistance by hydrolyzing cell wall polysaccharides of *Vd* and activating plant immune pathways. Natural variation contributes to a differential CCAAT/CCGAT elements in the *OSM1* promoter in cotton accessions. All *G. hirsutum* (*Gh*) exhibit the CCAAT haplotype, while there are two haplotypes of CCAAT/CCGAT in *G. barbadense*, with higher expression and stronger VW resistance in CCGAT haplotype. A NFYA5 transcription factor binds to the CCAAT element of *GhOSM1* promoter and inhibits its transcription. Silencing *GhNFYA5* results in higher *GhOSM1* expression and enhances VW resistance. These results broaden the insights into the functional mechanisms of osmotin and provide an effective strategy to breed VW‐resistant cotton.

## Graphical abstract

Osmotin OSM1 confers Verticillium wilt (VW) resistance by hydrolyzing cell wall polysaccharides of *Verticillium dahliae* (*Vd*) and activating plant immune pathways. A natural A/G variation in the *OSM1* promoter produces two haplotypes of CCAAT/ CCGAT, and causes the differential transcription of *OSM1*. In susceptible cotton accessions, transcription factor NFYA5 directly binds to the CCAAT element of the *OSM1* promoter and represses *OSM1* expression, resulting in susceptibility to *Vd*. However, in resistant cotton accessions, NFYA5 binding efficiency in CCGAT element is significantly decreased, thus *OSM1* is expressed at high levels and is rapidly induced by *Vd*, exhibiting enhanced VW resistance.

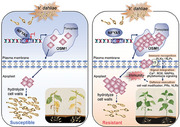

## 1 Introduction

Verticillium wilt (VW) is caused by the soil‐borne vascular fungus, *Verticillium dahliae* Klep. (*Vd*). The *Vd* has a broad host range and can cause severe damage to crops such as cotton, tomato, and potato.^[^ ^1^ ^]^ The *Vd* survives in the soil for a long time in the form of microsclerotia, which infects the plant roots directly under suitable conditions. First, it penetrates the root epidermis to colonize the vascular system in the xylem, then blocks the vessel and releases toxins.^[^ ^2^, ^3^ ^]^ VW leads to leaf chlorosis, wilting, and plant death, significantly reducing crop yield and quality. Because *Vd* spreads in the soil and colonizes the interior of vascular tissue, using chemical fungicides to control VW is inefficient and environmentally harmful.^[^ ^4^ ^]^ Thus, the most reasonable control approach is to mine essential resistance genes and breed VW‐resistant plant cultivars.

Cotton (*Gossypium* spp.) is an important natural textile fiber crop worldwide. The tetraploid *Gossypium hirsutum* and *G. barbadense*, originated from a single hybridization event between A‐ and D‐diploid species, are two main cotton cultivated species. Most *G. barbadense* accessions have resistance to VW, while *G. hirsutum*, constituting ≈97% of cotton production worldwide, is absent for the resistance.^[^ ^5^ ^]^ Resistance to VW in cotton is a complex genetic trait controlled by multiple loci and susceptible to environmental influences.^[^ ^6^ ^]^ To date, more than 400 VW resistance‐associated quantitative trait loci (QTLs) have been detected using marker‐assisted mapping or genome‐wide association studies, and they are widely distributed in almost all of the 26 tetraploid cotton chromosomes.^[^ ^7^, ^8^, ^9^, ^10^, ^11^ ^]^ However, only a few of these genes have been cloned and functionally characterized. Map‐based cloning revealed that a nonsense mutation within the coding region of *GhLMMD* in cotton led to the overaccumulation of 5‐aminolevulinic acid, which elevated levels of reactive oxygen species and salicylic acid, and promoted the activation of immune response and enhancement of VW resistance.^[^ ^12^ ^]^ By resolving structural variations in the genomes of *G. barbadense* and *G. hirsutum*, variations associated with VW resistance were reported to mainly locate in the D subgenome, while those for fiber yield traits were largely in the A subgenome. Further studies indicated that the pathogenesis‐related 10/Bet v1 protein family gene *GhNCS* in chromosome D11 was a plausible causal gene controlling VW resistance.^[^ ^13^ ^]^ However, due to the escalating threat posed by cotton VW to production, and the limited effects of individual loci on VW resistance, it is imperative to isolate more VW resistance genes, especially those that could enhance resistance without adversely affecting other agronomic traits in cotton.

Plants have evolved two major defense systems, PTI (Pathogen‐associated molecular patterns‐triggered immunity) and ETI (Effector‐triggered immunity), in response to pathogen attacks.^[^ ^14^ ^]^ PTI systematically defends against pathogen invasion, whereas ETI often leads to localized programmed cell death.^[^ ^15^ ^]^ These two processes activate many similar downstream responses, including hormone accumulation, the production of reactive oxygen species (ROS), and the expression of defense‐related proteins.^[^ ^16^, ^17^ ^]^ The secretion of defense‐related proteins into the extracellular space is crucial for establishing host plant resistance. This process directly and effectively controls pathogen invasion to the maximum extent. Among them, some proteins encoded by pathogenesis‐related genes (PRs), such as PR1, PR2, and PR5 exhibit antimicrobial activities, strongly suggesting their defensive roles.^[^ ^18^, ^19^ ^]^ PRs are often defined as proteins that are either absent or present only in minimal quantities in healthy tissues but accumulate significantly under pathological conditions in the presence of pathogens, and they serve as important biochemical markers to differentiate the initiation of plant disease resistance response.^[^ ^20^, ^21^ ^]^ Nevertheless, the essential PRs and their functional mechanisms during defense against pathogens in cotton remain enigmatic.

PR5s are a class of cysteine‐rich proteins, also known as thaumatin‐like proteins (TLPs) due to their sequence similarity to thaumatin proteins.^[^ ^22^ ^]^ The 16 conserved cysteine residues are evenly distributed throughout the PR5s, forming 8 disulfide bonds to safeguard proteins’ accurate folding and stability. This structural feature also enhances their ability to withstand protease enzymes, pH variations, and heat‐induced denaturation.^[^ ^23^, ^24^ ^]^ One reported PR5 protein with 244 residues, which lacks a sweet taste, accumulates during pathogen infection. Also, it is produced during osmotic stress in tobacco cells and is therefore also known as osmotin, which enhances tolerance against various biotic or abiotic stresses in plants.^[^ ^25^, ^26^ ^]^ Significant amounts of osmotin have been found in root and stem tissues,^[^ ^27^ ^]^ and they exhibit antifungal activity by causing spore lysis, reducing spore viability, and inhibiting spore germination and hyphal growth.^[^ ^28^ ^]^ The five highly conserved amino acids REDDD dispersed in the primary sequence can form a conserved acidic cleft domain at the 3D level, ensuring the antifungal activity of osmotin.^[^ ^29^ ^]^ Several studies have noted that osmotin enters the plasma membrane, leading to transmembrane pore formation and membrane rupture, which may be related to the recognition of membrane receptors.^[^ ^30^, ^31^ ^]^ *OsOSM1*, encoding an osmotic protein in rice, is strongly induced by *Rhizoctonia solani* in sheath blight‐resistant rice varieties but not in susceptible varieties. Overexpression of *OsOSM1* improves disease resistance in rice.^[^ ^32^ ^]^ Potatoes overproducing osmotin protein show increased resistance to *Phytophthora infestans*, and the purified proteins in vitro are more effective against the pathogen.^[^ ^33^ ^]^ TcOSM1 from *Theobroma cacao* inhibits the growth of *Moniliophthora perniciosa* mycelium and spore germination of the phytopathogenic fungi *Fusarium* f. sp. glycines and *Colletotrichum gossypi* in vitro.^[^ ^34^ ^]^ Despite these advances on osmotin genes of different plants, the role of osmotin and the regulatory mechanisms involved in VW resistance have remained largely unknown in cotton.

In the previous report, our group used *G. barbadense* acc. Hai7124 and *G. hirsutum* cv. Junmian1, which showed resistance and susceptibility to *Vd*, respectively, to produce (Junmian1 × Hai7124) × Junmian1 BC_1_ segregating population, and identified a major QTL locus associated with cotton VW resistance on chromosome D11.^[^ ^35^ ^]^ In this study, 28 pairs of SSR primers were further applied to fine map the QTL interval to 1.58 Mb. Combined with RNA‐seq data of *Vd*‐induced roots of cotton seedlings of *G. barbadense* acc. Hai7124 and *G. hirsutum* acc. TM‐1, a vital disease‐resistance gene *GbOSM1* was identified. We confirmed that *GbOSM1* played an important role in inhibiting the growth of *Vd* and triggering the overall immune response in plants. A natural A/G polymorphism in *GbOSM1* promoter of different cotton accessions formed two haplotypes of CCAAT and CCGAT elements, led to the changed binding efficiency of a NFYA5 transcription factor, further influenced the *GbOSM1* transcripts and plant disease resistance. This study expands our insights of osmotin proteins in plants against pathogens, also provides essential gene resources for breeding VW‐resistant cultivars in cotton.

## 2 Results

### 2.1 *GbOSM1* is a Newly Identified VW Resistance Gene in Cotton

**Figure 1:**
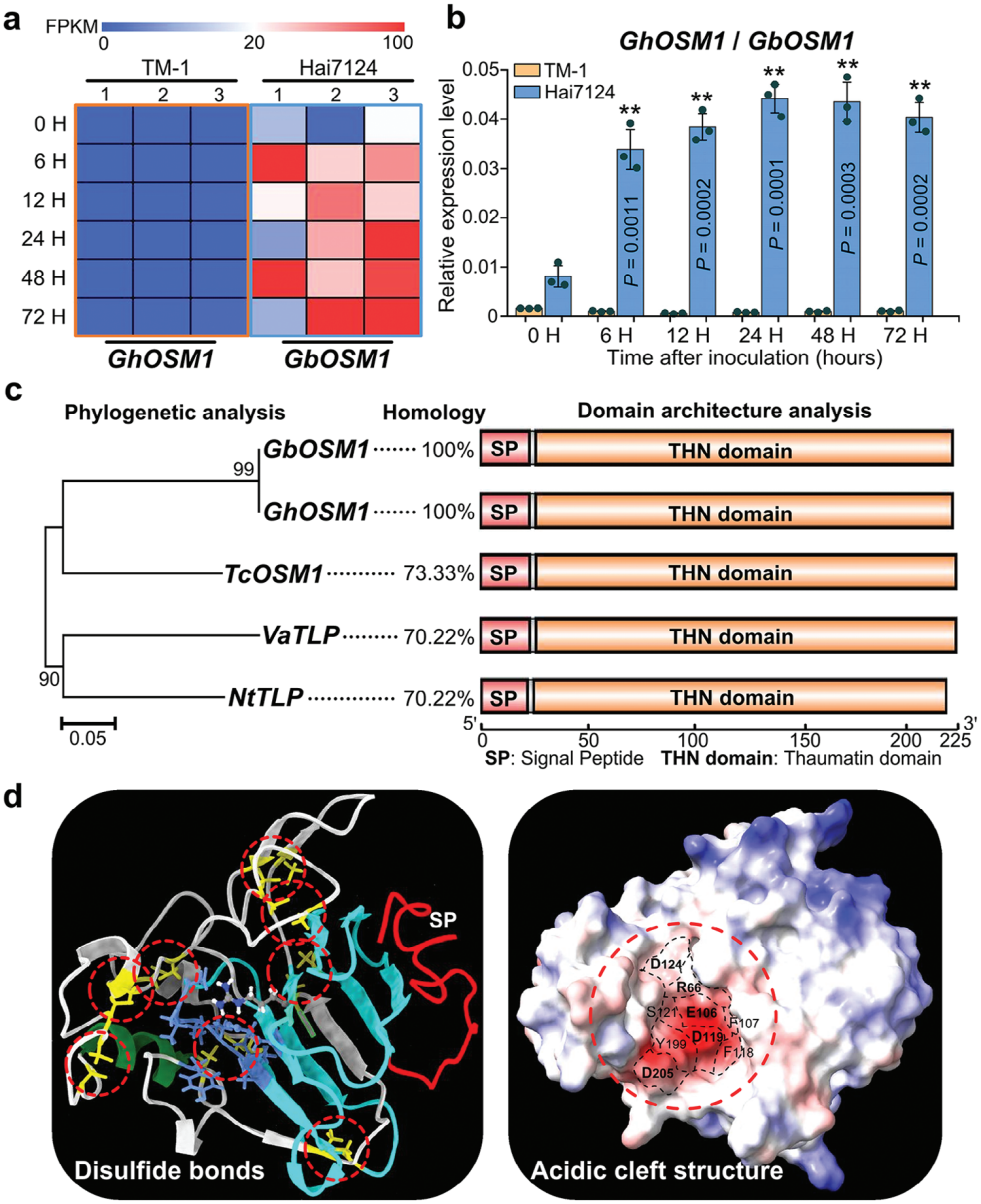
Expression and protein structure analysis of *OSM1* genes. a) *GhOSM1/GbOSM1* expression pattern in roots of TM‐1 and Hai7124 seedlings in response to *Vd* infection. The expression data were FPKM values used to calculate the expression level of *GhOSM1/GbOSM1*. Colored squares indicated expression levels from 0 (blue) to 100 (red). 1, 2, and 3 represent three biological replicates. Expression patterns were visualized using MeV 4.7.0. *Gh*, *G. hirsutum*; *Gb*, *G. barbadense*. b) RT‐qPCR analysis of *OSM1* expression in response to *Vd* infection. Error bars represent the standard deviation of three independent biological replicates for each experiment. The statistical analyses were performed by comparing expression levels at different time points between *Vd* infection and mock treatment using Student's *t*‐test (**P < 0.01). c) The phylogenetic classification and structural analysis of OSM1 homologs in different plant species. A phylogenetic tree was generated using the maximum likelihood method with the WAG model in MEGA v5.1 (http://www.megasoftware.net/), and the reliability of interior branches was assessed with 1000 bootstrap re‐samplings. DNAMAN software (http://www.lynnon.com/) was used to compare homology between protein sequences. The domains of OSM1 were analyzed using SMART (http://smart.embl.de) and INTERPROSCAN software (http://www.ebi.ac.uk/interpro/). *Tc*, *Theobroma cacao*; *Va*, *Vitis amurensis*; *Nt*, *Nicotiana tabacum*. d) The predicted structure of GbOSM1 (PDB ID: 1Z3Q) was displayed as a diagram and surface views using UCSF Chimera software. The red circle shows 16 conserved cysteines forming eight disulfide bonds, and the acidic cleft structure, REffDsDyD.

*G. barbadense* and *G. hirsutum* share a common allotetraploid ancestor. *G. barbadense*, represented widely by the Hai7124 accession, typically exhibits greater resistance to VW compared to *G. hirsutum*, as represented by the TM‐1 accession (Figure S1, Supporting Information). To mine the key disease resistance‐related gene in *G. barbadense*, a major QTL associated with cotton VW resistance on chromosome D11 was identified using the BC_1_ segregating population of *G. barbadense* and *G. hirsutum* in our previous study.^[^ ^35^ ^]^ Here, we further added 15 SSR loci and fine mapped the QTL interval to 1.58 Mb, explaining 11.66% to 13.25% of the total phenotypic variance (Figure S2a, Supporting Information). This QTL interval contains 95 genes. We found that many proteins encoded by these genes were related to plant disease resistance, such as TIR‐NBS‐LRR proteins, disease resistance family proteins, receptor‐like proteins, and pathogenesis‐related protein (osmotin). Combined with RNA‐seq data of *Vd*‐induced roots of cotton seedlings of Hai7124 and TM‐1,^[^ ^36^ ^]^ a *Osmotin1‐like* gene (*GB_D11G3398* in Hai7124 and *GH_D11G3351* in TM‐1, named *GbOSM1* and *GhOSM1*, respectively) raised our special concerns because its expression was at a high level in the Hai7124 roots, with substantially up‐regulated expression after *Vd* inoculation. In contrast, it was hardly expressed in the roots of *G. hirsutum* acc. TM‐1 (**Figure** **1**a; Figure S2b, Supporting Information). The RT‐qPCR experiments yielded the same results (Figure 1b).

Phylogenetic analysis showed that *OSM1* homologs from different cotton species were highly conserved and had higher homology to *TcOSM1*, *VaTLP* and *NtTLP* in *Theobroma cacao, Vitis amurensis* and *Nicotiana tabacum*, respectively, while the *OSM1* did not cluster with the previously reported cotton *GhPR5*/*GhOSM34*, indicating it is a newly identified osmotin encoding gene related to plant disease resistance (Figure S3, Supporting Information). The tissue organ expression showed that *OSM1* was characterized by exceptionally high expression in roots and stems of Hai7124, whereas it was not expressed in almost all tissues of TM‐1 (Figure S4, Supporting Information).

OSM1 has a large conserved Thaumatin domain, constituting ≈90% of the total amino acid sequence length. This domain shows high sequence similarity among different plants, but exhibits low signaling peptide identity at the C‐terminal (Figure 1c; Figure S5, Supporting Information). The 16 conserved cysteine residues in the Thaumatin domain form 8 stable disulfide bonds, ensuring the structural stability of the protein. Also, GbOSM1 has an acidic cleft structure of REffDsDyD, which is related to defense against foreign organisms (Figure 1d).

### 2.2 *GbOSM1* Positively Regulates the Resistance of Cotton to *Vd*

**Figure 2:**
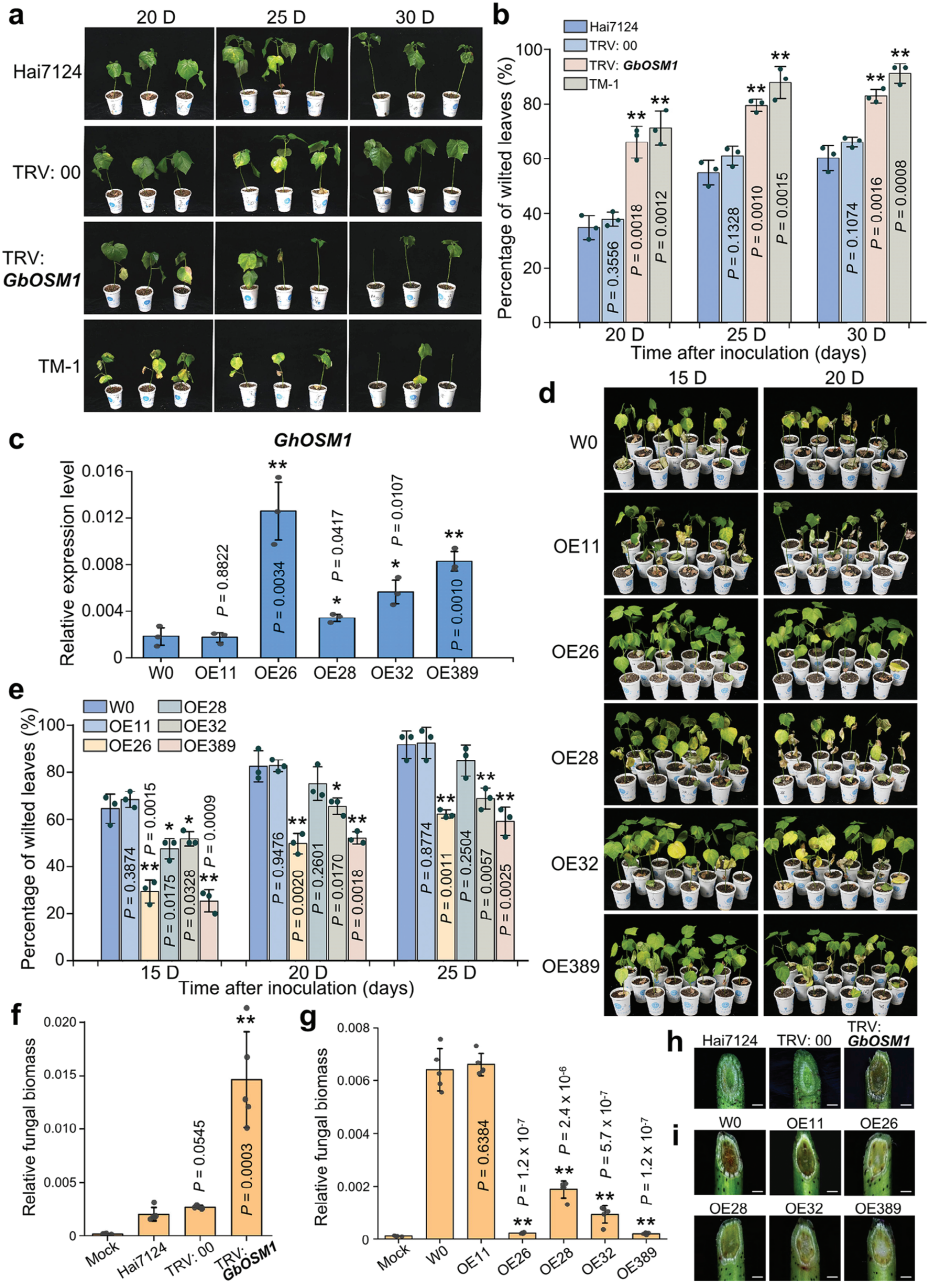
*GbOSM1* positively regulates VW resistance in cotton. a) Disease symptoms of *GbOSM1*‐silenced cotton plants were observed at 20, 25, and 30 days after *Vd* inoculation. b) Percentage of wilted leaves in *GbOSM1*‐silenced cotton plants after *Vd* inoculation. Each biological repeat contains at least 30 seedlings. Error bars represent the standard deviation of three biological replicates. Statistical analyses were performed by comparing with Hai7124 using Student's *t*‐test (**P < 0.01). c) RT‐qPCR analysis of *GbOSM1* transcripts level in five independent *GbOSM1* transgenic lines. Cotton histone 3 (AF024716) serves as the internal control. W0, wildtype cotton; OEs represent the different transgenic cotton lines, respectively. OE11, a negative control obtained during tissue culture. Error bars represent the standard deviation of three biological replicates. Statistical analyses were performed by comparing with W0 using Student's *t*‐test (*P < 0.05, **P < 0.01). d) *GbOSM1* overexpression enhances Verticillium wilt resistance in cotton. Four *GbOSM1* overexpression transgenic lines named OE26, OE28, OE32, and OE389 were used for disease resistance analysis. The wildtype cotton W0 and OE11 with unchanged *GbOSM1* transcripts were used as controls. Photographs were taken at 15 and 20 days post‐*Vd* inoculation. e) Percentage of wilted leaves in *GbOSM1* overexpression transgenic cotton plants and controls after *Vd* inoculation. Statistical analyses were performed by comparing with W0 using Student's *t*‐test (*P < 0.05, **P < 0.01). f,g) qPCR analysis of fungal biomass in *GbOSM1‐*silenced, overexpressed, and control plants. The DNAs of stems were extracted from plants at 10 days after *Vd* inoculation, respectively. The mock plants were Hai7124 or W0 without *Vd* infection. Error bars represent the standard deviation of five biological replicates. Statistical analyses were performed by comparing with controls using Student's *t*‐test (*P < 0.05, **P < 0.01). h,i) Vascular discoloration was observed in *GbOSM1*‐silenced and overexpressed plants compared to the controls after inoculation with *Vd*. Photographs were taken with a stereoscope (Olympus MVX10, Tokyo, Japan) at 10 days after inoculation. Scale bars: 1.5 mm.

To elucidate the role of *GbOSM1* during cotton defense against *Vd*, we utilized the virus‐induced gene silencing (VIGS) technique to silence *GbOSM1* in Hai7124, with TRV: 00 as the mock treatment and TRV: *GbCLA1* as the positive control. At two weeks after infiltration with the TRV: *GbCLA1* construct, cotton leaves showed obvious photobleaching, and the decreased expression level of *GbOSM1* was confirmed through RT‐qPCR analysis (Figure S6, Supporting Information). The cotton seedlings were then inoculated with the *Vd* strain V991. The TRV: *GbOSM1* plants exhibited more etiolation, wilting, and even abscission of leaves than TRV: 00 and Hai7124 plants (**Figure** **2**a). At 25 days post‐inoculation, the percentage of wilted leaves was ≈61% for TRV: 00 and 55% for Hai7124 plants, while the TRV: *GbOSM1* plants showed 80% wilted leaves (Figure 2b). In addition, due to the low expression level of *GhOSM1* in *G. hirsutum* acc. TM‐1, there was no obvious difference of disease resistance detected between TRV: 00 and TRV: *GhOSM1* plants, indicating that the high expression level of *OSM1* contributes to resistance against VW in cotton (Figure S7, Supporting Information).

In order to further clarify the function of *GbOSM1*, we performed overexpression of *GbOSM1* in *G. hirsutum* acc. W0, and fourteen independent transgenic lines were obtained (Figure S8a, Supporting Information). Via RT‐qPCR analysis, four independent transgenic lines with different expression levels (OE26, OE28, OE32, OE389) and one negative line (OE11) were selected for further studies (Figure 2c; Figure S8b, Supporting Information). After *Vd* inoculation, a more resistant phenotype was observed in the *GbOSM1* transgenic line, with less yellowing, wilting, and necrosis in the leaves. At 15 days after infection, the percentage of wilted leaves in W0 and OE11 plants reached 64% and 68%, respectively. In contrast, the OE26, OE28, OE32, and OE389 plants showed a lower percentage of wilted leaves at 29%, 48%, 52%, and 25%, respectively (Figure 2d,e). The expression level of *GbOSM1* was positively correlated with the percentages of wilted leaves in transgenic plants (Figure S9, Supporting Information).

Fungal biomass assays and observation of browning in vascular bundles further confirmed that more fungi accumulated in the stems of *GbOSM1*‐silenced plants (Figure 2f,h). In contrast, the fungal biomass in *GbOSM1* transgenic lines was lower than that of control plants (Figure 2g,i).

### 2.3 GbOSM1 Inhibits the Growth of *Vd* by Hydrolyzing Polysaccharides of the Fungal Cell Wall

**Figure 3:**
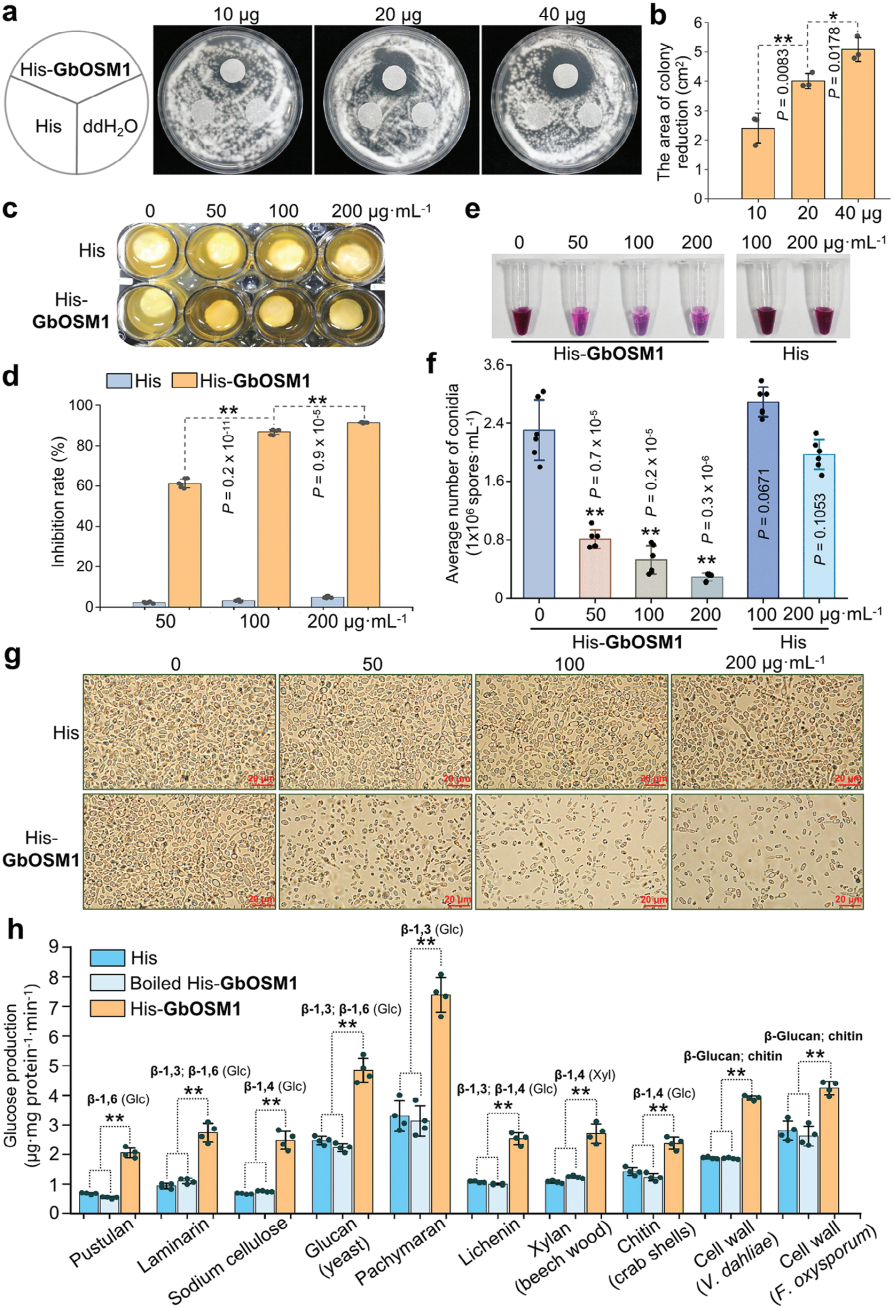
GbOSM1 hydrolyzes the *Vd* cell wall, inhibits mycelial growth and spore production. a) The filter papers were placed on the PDA medium containing *Vd* spores, then treated with 10 µg, 20 µg, 40 µg of His‐GbOSM1, His proteins, and sterile water, respectively. The photograph was taken at 4 days after incubation. b) The area of colony reduction in a) was measured using ImageJ. Error bars represent the standard deviation of three biological replicates. Statistical analyses were performed using Student's *t*‐test (*P < 0.05; **P < 0.01). c) The effect of His‐GbOSM1 and His proteins on the mycelial growth of *Vd*. In 24‐well tissue culture plates containing 4 × concentrated potato dextrose broth (200 µL), *Vd* mycelia were added. Subsequently, the wells were treated with proteins at concentrations of 0, 50, 100, and 200 µg mL^−1^. The mycelia were cultured for an additional 5 days and photographed. d) Analysis of the inhibition rate of His‐GbOSM1 and His proteins on the mycelial growth of *Vd*. Error bars represent the standard deviation of six biological replicates. Statistical analyses were performed using Student's *t*‐test (**P < 0.01). e) The CPK medium containing 1  ×  10^4^ conidia mL^−1^ of *Vd* was added with different concentrations of proteins. The number of conidium werwasen observed using Periodic Acid‐Schiff staining after being cultured for 3 days. f) The number of conidia in e) was counted using a hemocytometer plate. Error bars represent the standard deviation of six biological replicates. Statistical analyses were performed using Student's *t*‐test (**P < 0.01). g) The density of conidia in f) was observed under a microscope. Scale bars: 20 µm. h) The purified His‐GbOSM1 protein was used in enzyme assay with various substrates, including *V. dahliae* and *F. oxysporum* cell wall component, Chitin from crab shells, Xylan from beech wood, Glucan from yeast, Pustulan, Laminarin, Sodium cellulosate and Lichenin. His protein and boiled His‐GbOSM1 were used as the negative controls, respectively. Enzymatic activity was designated as micrograms of glucose produced per minute per milligram of protein. Error bars represent the standard deviation of four biological replicates. Statistical analyses were performed using Student's *t*‐test (**P < 0.01).

To conduct in vitro antifungal assays, the GbOSM1 recombinant protein was expressed and purified. The molecular masses of the His and His‐*GbOSM1* purified proteins were determined using SDS‐PAGE. The target proteins were detected using anti‐His antibodies (Figure S10, Supporting Information). In the PDA solid medium for culturing *Vd*, a higher level of GbOSM1 protein inhibited the growth of fungi more effectively (**Figure** **3**a,b). Similarly, GbOSM1 protein inhibited hyphal growth in the PDB liquid culture medium (Figure 3c,d). In addition, we added various concentrations of GbOSM1 protein to a *Vd* spore suspension containing 1 × 10^4^ conidia mL^−1^. After culturing for three days, it was confirmed that the GbOSM1 protein significantly inhibited the production of fungal conidia as demonstrated by Periodic Acid‐Schiff staining (Figure 3e), hemocyte counting method (Figure 3f), and microscopic observation (Figure 3g). These results indicate a dose‐dependent antifungal activity of GbOSM1 against *Vd*.

In order to investigate the mechanisms by which GbOSM1 acts against *Vd*, the *Vd* treated with His and His‐GbOSM1 proteins were collected (Figure S11a, Supporting Information). The molecular basis of the effect of *GbOSM1* on *Vd* was explored through RNA‐seq (Figure S11b, Supporting Information). However, only a few differentially expressed genes (DEGs) were enriched, suggesting that most of the genes in *Vd* are unaffected by GbOSM1 (Figure S11c, Supporting Information). The main components of fungal cell walls are chitin (a β‐1,4‐linked polysaccharide) and glucan (a β‐1,3/1,6‐linked polysaccharide).^[^ ^37^ ^]^ We further detected the hydrolytic activities of GbOSM1 on different glycosidic bonds in various substrates, and found that GbOSM1 could hydrolyze the β‐1,3/1,4/1,6‐bond to release glucose (Figure 3h; Figure S12, Supporting Information), indicating that GbOSM1 destroys cells by directly hydrolyzing various polysaccharide components of the fungal cell wall.

### 2.4 GbOSM1 Localizes to the Tonoplast and is Secreted Extracellularly in Response to Osmotic Stress or *Vd* Infection

**Figure 4:**
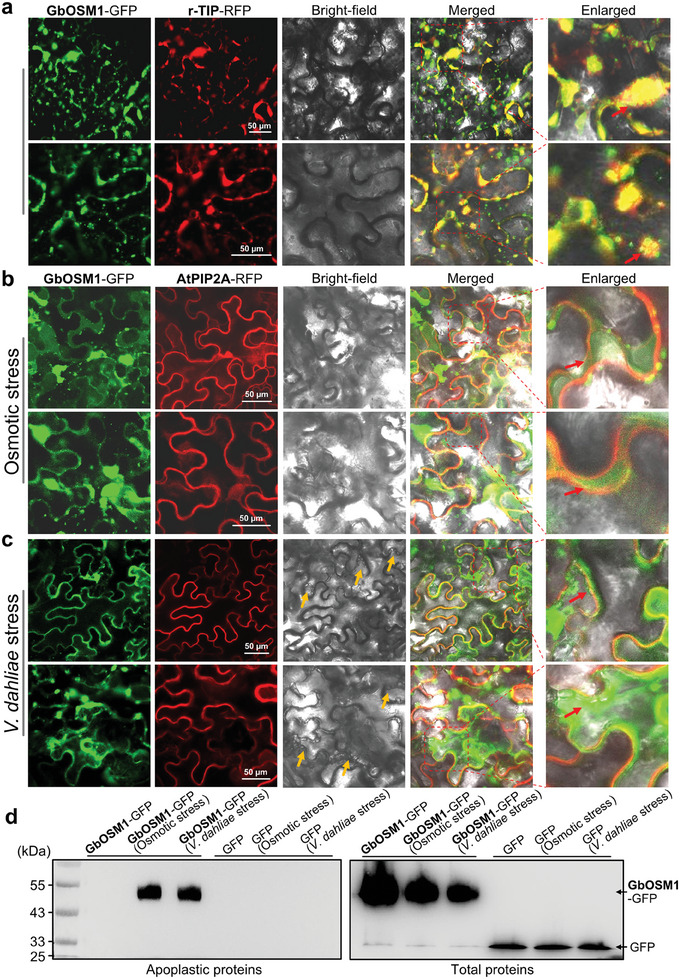
GbOSM1 protein is stored in the tonoplast, and secreted to the apoplast to perform a defensive role under osmotic or *Vd* stress. a) The GbOSM1‐GFP fusion co‐localizes with a tonoplast marker, r‐TIP‐RFP (aquaporin of the vacuolar membrane). Red arrow indicates the distribution of GbOSM1‐GFP green fluorescence in small vesicles. b) After treatment with 0.8 M mannitol solution, GhOSM1‐GFP was secreted into the apoplast. Red arrow indicates the apoplast space. c) After *Vd* infection, GhOSM1‐GFP accumulates in large quantities at the plasma membrane. Yellow arrows indicate infiltrated *Vd* can be observed in the bright field, and red arrow indicates the GbOSM1‐GFP green fluorescence overlapping the plasma membrane. Images were captured by confocal microscopy (LSM 780; Zeiss). Scale bars: 50 µm. d) Immunodetection of GbOSM1‐GFP and GFP in crude leaf protein extracts using a GFP antibody. Osmotic stress treatment was simulated by treating leaves with 0.8 M mannitol for four hours. After two days of Agrobacterium infestation, the induction treatment was carried out by injecting a *Vd* spore suspension (1 × 10^5^ conidia mL^−1^) at the exact location. Tobacco leaves were immersed in PBS buffer and subjected to vacuum treatment. The proteins secreted into the apoplast were obtained by centrifuging the leaves at high speed.

Based on the predicted subcellular localization, the GbOSM1 is primarily involved in the extracellular secretory pathway (Table S1, Supporting Information). The GbOSM1‐EGFP fusion protein was transiently expressed under the control of the 35S promoter in Arabidopsis protoplasts. Confocal microscopy showed that the GbOSM1‐EGFP‐dependent green fluorescence signal delineated a sharp closed circle, corresponding to the tonoplast (Figure S13, Supporting Information). Furthermore, the subcellular localization in *N. benthamiana* leaf epidermis indicated that GbOSM1‐GFP co‐localized with the tonoplast marker protein and exhibited a punctate distribution in small vesicle structures (**Figure** **4**a). Although some peroxisomal marker protein‐RFP signals were also distributed near the tonoplast, they did not overlap with the GbOSM1‐GFP signal (Figure S14a, Supporting Information). In addition, the GbOSM1‐GFP signal was distributed near the plasma membrane (Figure S14b, Supporting Information). When leaves treated with a 0.8 M mannitol solution to simulate osmotic stress, the GbOSM1 proteins were secreted into the apoplast (Figure 4b). After two days of *Agrobacterium* infiltration, we infected tobacco leaves with *Vd*, the GbOSM1‐GFP signal was also observed to accumulate substantially at the plasma membrane (Figure 4c). Dynamic observations showed that the intracellular transport of GbOSM1‐GFP was accelerated after *Vd*‐infected cells (Movies S1 and S2, Supporting Information). Further isolation of the apoplastic proteins from *N. benthamiana* leaf cells detected the GbOSM1‐GFP protein after inducing plasmolysis or infecting with *Vd*. In contrast, the GFP protein used as a control could not be detected in apoplast (Figure 4d). The results indicate that the GbOSM1 is stored in the tonoplast, and secreted from the plasma membrane into the apoplast to perform a defensive role under osmotic or *Vd* stress.

### 2.5 Overexpression of *GbOSM1* Activates Plant Immune Response

**Figure 5:**
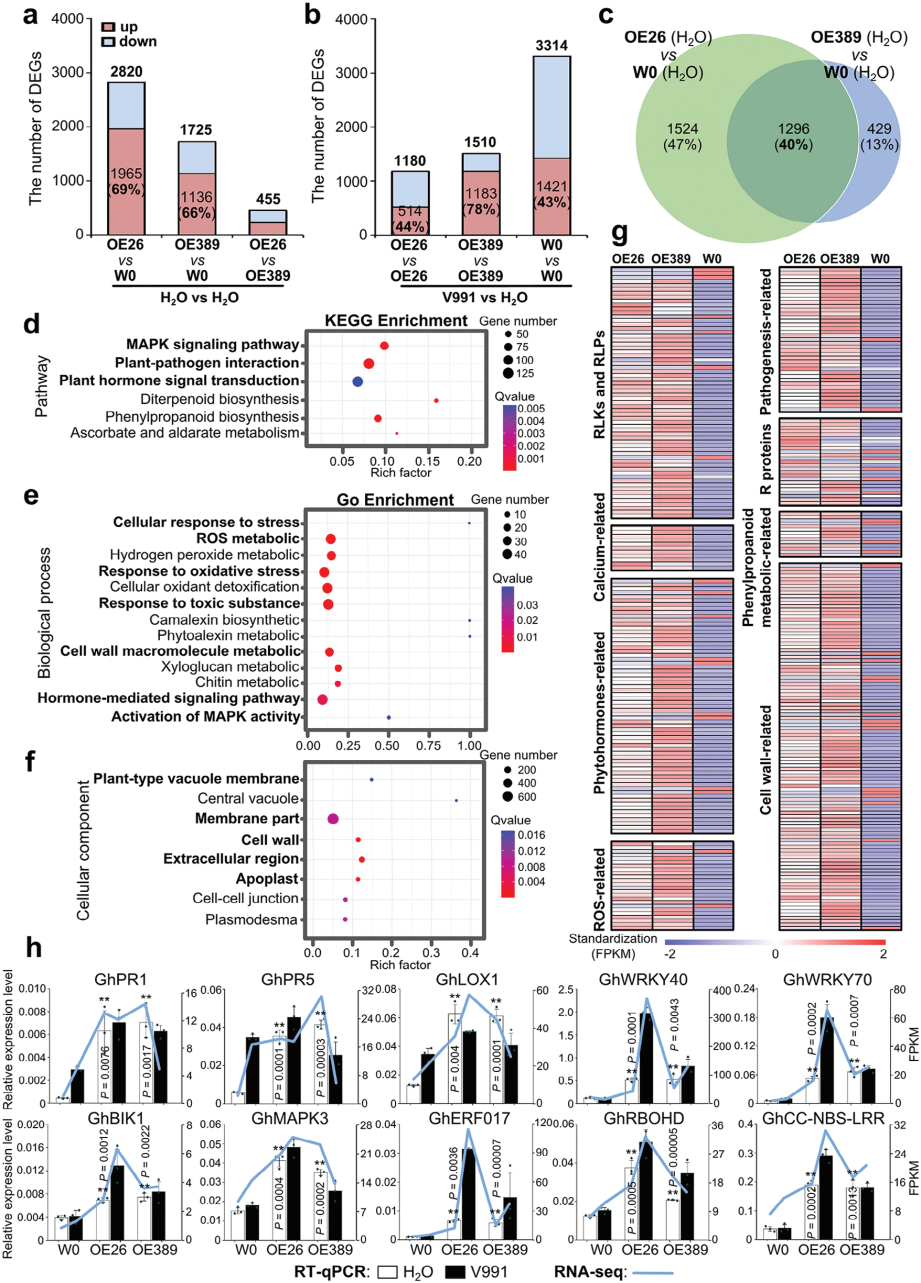
Overexpression of *GbOSM1* activates immunity‐related pathways. a,b) The number of differentially expressed genes (DEGs) in the roots of OE26, OE389, and W0 plants after three days of treatment with H_2_O or *Vd*. The red represents the number of up‐regulated DEGs, while the blue represents the number of down‐regulated DEGs in the histogram. c) The DEGs analysis for OE26 and OE389 with the wildtype cotton W0 under H_2_O‐treated conditions. Venn diagrams show that the two transgenic lines have mostly overlapped DEGs compared to W0. d–f) KEGG and GO enrichment analysis of 1296 DEGs between *GbOSM1* transgenic cotton plants OE26 and OE389 compared to W0. The false discovery rate adjusted the *q*‐value to 0.05. Rich factor: Percentage of enriched genes compared with the background in corresponding GO and KEGG terms. g) Heatmap illustrating the of differential expression of immune‐related genes in the *GbOSM1* transgenic line compared to non‐transgenic control plants. The numerical values for the blue‐to‐red gradient bar represent the FPKM standardization values of the DEGs in each sample. h) The expression of immune‐related genes was validated by RT‐qPCR. The folded line indicates the FPKM value of the genes in the RNA‐seq data. Asterisks indicate statistically significant differences, as determined by the Student's *t*‐test (**P < 0.01).

To further analyze the molecular basis of *GbOSM1* to improve disease resistance in cotton, we collected root samples from *GbOSM1* overexpression transgenic plants (OE26 and OE389) treated individually with water and *Vd* for three days for RNA‐seq analysis, with W0 as control. The reliability of the sequencing data was confirmed by cluster dendrogram analysis of 18 samples and RT‐qPCR analysis of 19 genes (Figure S15, Supporting Information). RNA‐seq analysis showed that overexpression of *GbOSM1* resulted in a significant increase in up‐regulated DEGs when comparing transgenic plants with W0 under water treatment (*q* < 0.05 and a fold change > 2) (**Figure** **5**a). Furthermore, we compared the DEGs in the roots of *GbOSM1* transgenic lines and W0 plants with inoculated and non‐inoculated *Vd* treatment, and found that the number of DEGs had obvious differences. The roots of W0 were severely affected by the pathogen, with as many as 3314 DEGs detected. In contrast, the roots of the transgenic plants were minimally affected, with less than half of the DEGs in W0 (Figure 5b). This also indicates that the *GbOSM1* transgenic plants have a more robust adaptation to *Vd* stress.

Under water treatment, the 1296 DEGs in OE26 and OE389 overlapped when compared with W0 (Figure 5c). GO and KEGG enrichment analysis showed that these DEGs were related to immune‐related pathways, such as MAPK signaling, plant‐pathogen interaction, and plant hormone signal transduction (Figure 5d), as well as biological processes such as ROS metabolism, response to toxic substance, cell wall macromolecule metabolism, and so on (Figure 5e). In addition, the molecular functions associated with these DEGs were related to cellular components such as vacuole, membrane part, cell wall, and apoplast (Figure 5f; Table S2, Supporting Information). Among them, immunity‐related genes were significantly up‐regulated in transgenic lines compared to those in W0 plants (Figure 5g,h **;** Table S3, Supporting Information). In addition, compared with TRV: 00 plants, TRV: *GbOSM1* plant roots show few DEGs under water treatment. However, the activation of many immune‐related genes was suppressed upon infection by *Vd* (Figure S16, Supporting Information).

Activation of the immune response is often accompanied by local programmed cell death (PCD), which prevents the spread of the disease through an orderly response. After 4–5 days of *Agrobacterium* carrying the 35S: *GbOSM1‐GFP* construct infiltrated into *N. benthamiana* leaves, we observed an apparent PCD reaction in the leaves (Figure S17a, Supporting Information). Additionally, Trypan Blue staining revealed distinct cell death at the infected position (Figure S17b, Supporting Information). However, the cell death induced by GbOSM1 was not as severe as that caused by the apoptosis protein BAX or the pathogenic elicitor NLP1. It was more similar to the immune response triggered by the receptor protein CERK1 (Figure S17c,d, Supporting Information). Ion leakage analysis was used to quantify cell death, which was consistent with the observed cell death (Figure S17e, Supporting Information). The RT‐qPCR results showed that the expressions of immune response‐related genes *NbPR1*, *NbWRKY22*, *NbBAK1*, *NbMAPK3*, and *NbRBOHD* were significantly upregulated in leaves expressing *GbOSM1‐GFP* (Figure S17f, Supporting Information). Taken together, the results indicate that the *GbOSM1* overexpression activates immune‐related pathways in plants and triggers a mild local cellular PCD response, which may be related to the recognition of specific receptors.

### 2.6 A Natural A/G Variation in the *OSM1* Promoter Leads to the Differential Expression of *OSM1* and Plant Disease Resistance

**Figure 6:**
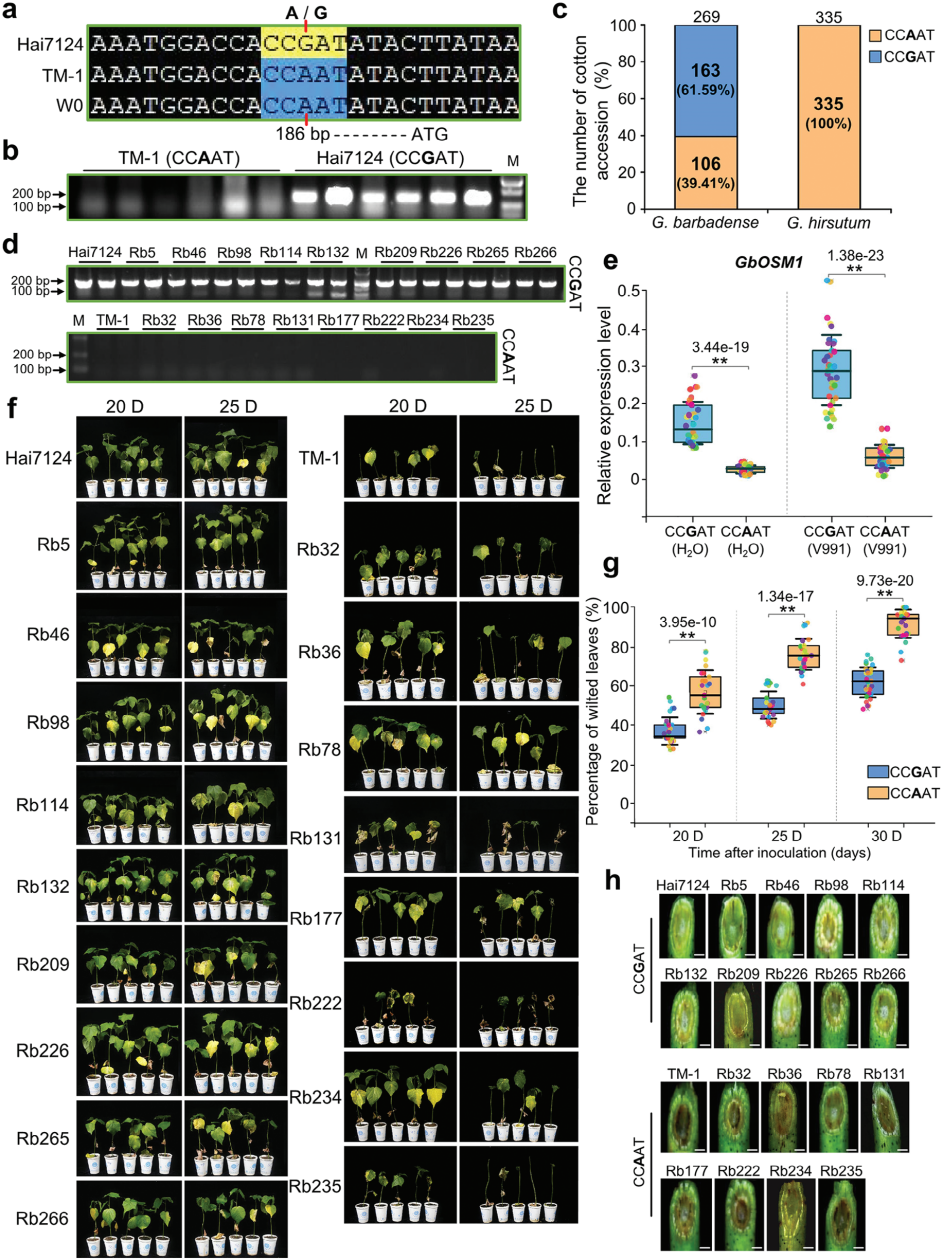
The A/G natural variation in the *OSM1* promoter contributes to differential transcription of *OSM1* and plant disease resistance. a) The A/G natural variation in the *OSM1* promoter was identified in *G. barbadense* acc. Hai7124, *G. hirsutum* acc. TM‐1, and transgenic receptor *G. hirsutum* acc. W0. A/G is located 186 bp upstream of ATG. b) SNAP marker was developed to detect A/G genotypes in the TM‐1 and Hai7124 genomes. M, molecular weight marker. c) Genotype detection of A/G variation in *G. barbadense* and *G. hirsutum* natural populations. Totally, 269 accessions in *G. barbadense* natural population and 335 accessions in *G. hirsutum* natural population, respectively. Yellow and blue represent the number of cotton accession with A and G haplotypes in the two sets of populations. d) PCR detection of A/G haplotypes in the representative *G. barbadense* accessions. Nine of the *G. barbadense* accessions have the G haplotypes, and eight have the A haplotypes. M, molecular weight marker. e) The *GbOSM1* expression level in the *G. barbadense* accessions carrying A/G haplotypes in d) was detected. The root tissue was extracted from three‐week‐old plants after treatment with *Vd* and H_2_O for three days, and RNA was extracted. Three biological replicates of roots were used for each *G. barbadense* accession. In box plots, different colors represent different *G. barbadense* accessions, center line indicates median, box limits denote upper and lower quartiles. Asterisks indicate statistically significant differences, as determined by the Student's *t*‐test (**P < 0.01). f) Disease symptoms of *G. barbadense* accessions in d) at 20 and 25 days after *Vd* inoculation. g) The percentage of wilted leaves of *G. barbadense* accessions at 20, 25 and 30 days after inoculation with *Vd*. Three biological repeats for each *G. barbadense* accession. Each biological repeat contains at least 30 seedlings. In box plots, different colors represent different *G. barbadense* accessions, center line indicates median, box limits denote upper and lower quartiles. Statistical analyses were performed using Student's *t*‐test (**P < 0.01). h) Vascular discoloration was observed in *G. barbadense* accessions with the G haplotypes compared to the *G. barbadense* accessions with the A haplotypes. Photographs were taken with a stereoscope (Olympus MVX10, Tokyo, Japan) at 10 days after inoculation. Scale bars: 1.5 mm.

To investigate the reasons for the expression difference of *OSM1* between *G. barbadense* acc. Hai7124 and *G. hirsutum* acc. TM‐1, we cloned the *OSM1* genomic sequence and the upstream 1371 bp promoter sequence in the two cotton accessions to detect the genetic variation of *OSM1* homologs. There were no differences in the *OSM1* genomic sequence between the two accessions, while three SNP polymorphism in their promoter sequence were detected. By searching the PlantCARE databases, a putative transcription start site was predicted at ‐25 bp upstream of the ATG of *OSM1* (Figure S18, Supporting Information). We found a A/G SNP variation at position ‐186 bp upstream of the ATG resulted in the two haplotypes of CCAAT/CCGAT elements (**Figure** **6**a), and developed SNP marker to effectively distinguish A/G variation between Hai7124 and TM‐1 (Figure 6b). Furthermore, we investigated the resequencing data of 335 *G. hirsutum* and 269 *G. barbadense* accessions, and found that all *G. hirsutum* accessions were CCAAT haplotype. In contrast, 60.59% of *G. barbadense* were CCGAT haplotype, and 39.41% were CCAAT haplotype (Figure 6c). The results reflect the prevalence of A/G polymorphism of the *OSM1* promoter in different cotton accessions.

To clarify the relationship between the variations in CCGAT/CCAAT elements, the expression levels of *GbOSM1*, and plant disease resistance, we randomly selected 17 *G. barbadense* accessions, 9 with CCGAT haplotype and 8 with CCAAT haplotype, for further analysis (Figure 6d). Compared with cotton accessions with CCAAT haplotype, the expression level of *GbOSM1* was higher in cotton with CCGAT haplotype, and the up‐regulated expression was more evident after induction by *Vd* (Figure 6e). In addition, the VW resistance of cotton accessions with CCGAT haplotype was significantly higher than that with CCAAT haplotype (Figure 6f,g), and the number of fungi invading the vascular tissue of stems was obviously less at the initial stage of *Vd* infection (Figure 6h). These results suggest that the difference in CCAAT/CCGAT elements cause differential transcription of *OSM1* and plant VW resistance.

### 2.7 NFYA5 Binds to the CCAAT Element and Inhibits *OSM1* Gene Expression

Nuclear factor Y (NF‐Y) transcription factors are important regulators during plant development and stress‐induced responses. Among them, NF‐YA can bind to the CCAAT element in the downstream target genes promoter region to regulate the expression of genes.^[^ ^38^ ^]^ There were 10 NFYA genes identified in *Arabidopsis*, while 32 NFYA genes were found in cotton based on the CBFB_NFYA domain (PF02045) in both Hai7124 and TM‐1. Among them, one gene named *NFYA5*, which is most similar to *AtNFYA5* in Arabidopsis, was significantly up‐regulated at different time points induced by *Vd* (Figure S19, Supporting Information). This up‐regulation expression was also consistent with the induced expression pattern of *GbOSM1*, and further verified by RT‐qPCR. NFYA5 shows low homology among different plants, except for the conserved CCAAT‐binding transcription factor domain. Subcellular localization showed that GhNFYA5 was a nuclear‐localized transcription factor (Figure S20, Supporting Information).

**Figure 7:**
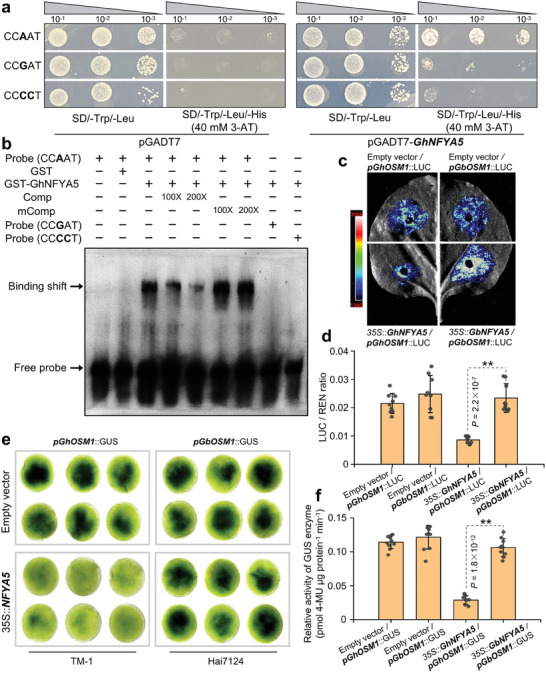
GhNFYA5 inhibits the expression of *GhOSM1* by directly binding to the CCAAT element. a) The yeast one‐hybrid assay shows that GhNFYA5 binds to the CCAAT element, but not to the CCGAT element. The CCCCT element with the base mutation is used as the negative control. 40 mM of 3‐amino‐1,2,4‐triazole (3‐AT) is applied to suppress the basal expression of the bait construct. b) EMSA assays suggest that the GhNFYA5 protein binds to the *GhOSM1* promoter (TM‐1) rather than the *GbOSM1* promoter (Hai7124) in vitro. The GST‐GhNFYA5 protein (2 µg) was incubated with the labeled probe. The unlabeled (Comp) probe or the mutated DNA (mComp) probe at 100 × and 200 × molar excess competed with the labeled probe. c) Transactivation analysis using the LUC reporter system reveals that the GhNFYA5 protein binds to the *GhOSM1* promoter and inhibits downstream LUC gene expression. The *GhOSM1/GbOSM1* promoter sequences in Hai7124 and TM‐1 are co‐transformed with GhNFYA5 and GbNFYA5, respectively. d) Dual‐LUC transient expression assay showed that GhNFYA5 inhibited the activity of *GhOSM1* promoter. Data were collected from three biological replicates for each reaction with three technical replicates for each. e) Transient GUS expression analysis confirms the binding of GhNFYA5 on the *GhOSM1* promoter and represses downstream gene expression. GUS staining of representative leaf pieces that were co‐infiltrated with the effectors and the reporters as indicated. f) GUS activity assays showed that GhNFYA5 inhibited the activity of *GhOSM1* promoter. Data were collected from five biological replicates for each reaction with two technical replicates for each. Asterisks indicate statistically significant differences, as determined by the Student's *t*‐test (**P < 0.01).

Next, to test whether the CCAAT/CCGAT elements associated with *OSM1* expression level, we performed a series of experiments to investigate the binding efficiencies of GhNFYA5 on the CCAAT/CCGAT elements. Yeast one‐hybrid assay demonstrated that GhNFYA5 binds to the CCAAT element but not to the CCGAT and CCCCT elements (mutated element) (**Figure** **7**a). Then, electrophoretic mobility shift assays (EMSA) were performed to investigate whether GhNFYA5 binds to the CCAAT element in the promoter region of *GhOSM1*. The results showed that GhOSM1 protein could not bind to biotin‐labeled CCGAT‐carrying and CCCCT‐carrying probes but could bind to the biotin‐labeled CCAAT‐carrying probe. Additionally, the addition of unlabeled probes (Competitive) significantly decreased the bindings. In contrast, adding competitive mutations does not affect its bindings (Figure 7b). Furthermore, in the firefly luciferase (LUC) assay, the *N. benthamiana* leaves infiltrated with GhNFYA5 effector and pGhOSM1 reporter (CCAAT haplotype) showed obviously weaker fluorescence intensity, while leaves infiltrated with GbNFYA5 effector and pGbOSM1 reporter (CCGAT haplotype) showed no distinct change compared with the control (Figure 7c). In addition, using the dual‐luciferase reporter system, the sequences of pGhOSM1 and pGbOSM1 were fused upstream of the LUC gene, respectively. The REN gene in the reporter construct was utilized as the internal control. The LUC/REN ratio was significantly reduced in *N. benthamiana* that co‐transformed with the GhNFYA5‐effector and pGhOSM1‐reporter (Figure 7d). Moreover, transient GUS expression analysis, including GUS staining and GUS activity analysis, also indicated that GhNFYA5 could bind to the *GhOSM1* promoter and inhibit its transcriptional level (Figure 7e,f).

**Figure 8:**
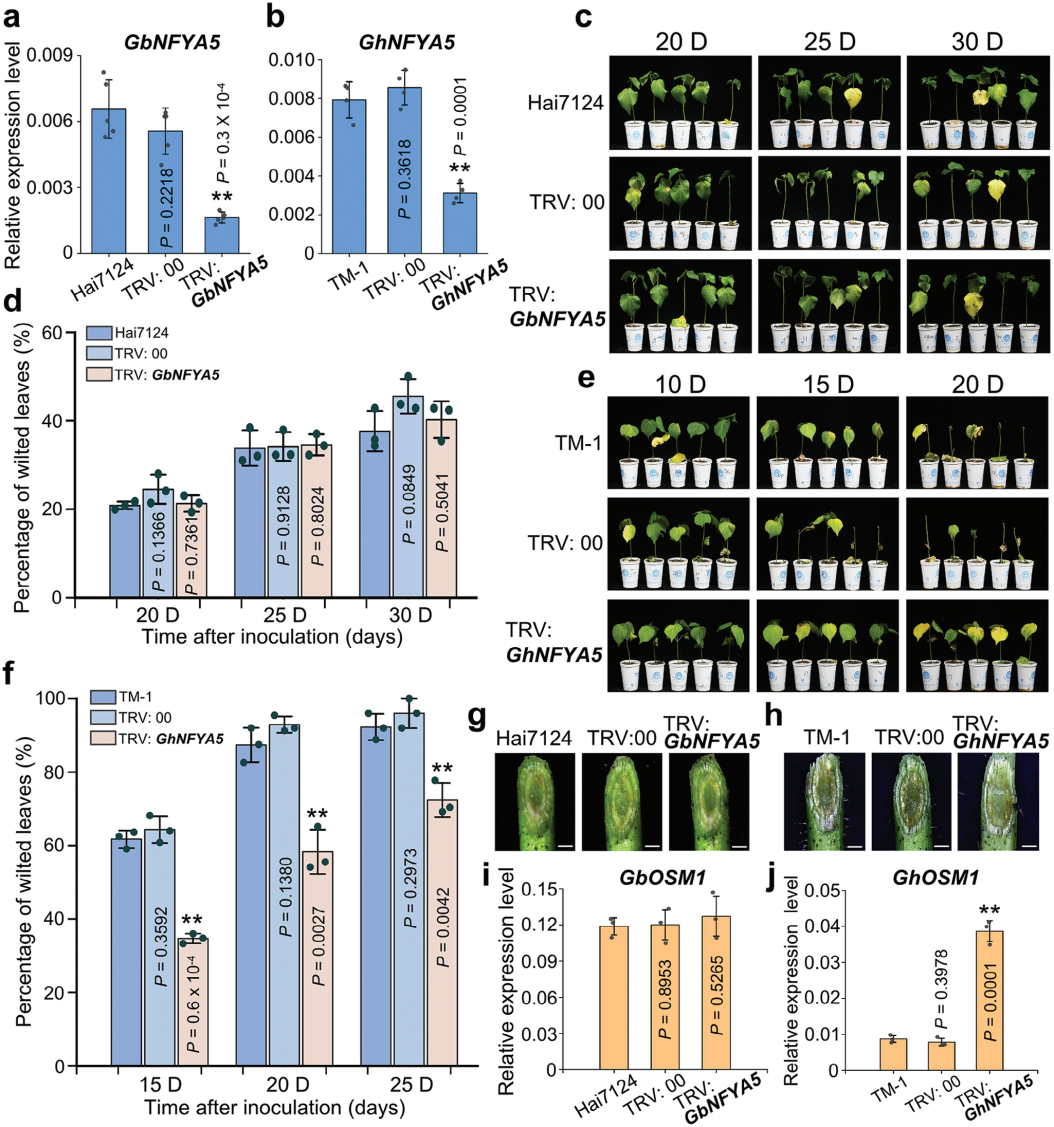
Silencing *GhNFYA5* activates *GhOSM1* transcripts level and enhances Verticillium wilt resistance in *G. hirsutum*. a,b) Verification of *NFYA5* silencing by RT‐qPCR in Hai7124 a) and TM‐1 b) VIGS plants. Error bars represent the standard deviation of five and four biological replicates, respectively. Statistical analyses were performed using Student's *t*‐test (**P < 0.01). c) Disease symptoms of *NFYA5‐*silenced cotton plants were observed at 20, 25, and 30 days after *Vd* inoculation in Hai7124 background. d) Percentage of wilted leaves in *NFYA5*‐silenced cotton plants after *Vd* inoculation in Hai7124 background. Each biological repeat contains at least 30 seedlings. Error bars represent the standard deviation of three biological replicates. e) Disease symptoms of *NFYA5‐*silenced cotton plants were observed at 20, 25, and 30 days after *Vd* inoculation in TM‐1 background. f) Percentage of wilted leaves in *NFYA5*‐silenced cotton plants after *Vd* inoculation in TM‐1 background. Each biological repeat contains at least 30 seedlings. Error bars represent the standard deviation of three biological replicates. Statistical analyses were performed using Student's *t*‐test (**P < 0.01). g‐h) Vascular discoloration in *NFYA5‐*silenced cotton plants compared with the controls after inoculation with *Vd* in Hai7124 g) and TM‐1 h) background, respectively. Photographs were taken with a stereoscope (Olympus MVX10, Tokyo, Japan) at 10 days after inoculation. Scale bars: 1.5 mm. i‐j) RT‐qPCR analysis of *GbOSM1/GhOSM1* in *NFYA5‐*silenced cotton plants and the controls in Hai7124 i) and TM‐1 j) background, respectively. Error bars represent the standard deviation of three biological replicates. Statistical analyses were performed using Student's *t*‐test (**P < 0.01).

To further investigate the role of NFYA5 in regulating *OSM1* expression and its function in disease resistance, we silenced the *NFYA5* expression in Hai7124 and TM‐1, respectively. As expected, the expression of *NFYA5* in both Hai7124 and TM‐1 roots was significantly inhibited after *Agrobacterium* carrying the TRV: *NFYA5* vector infection (**Figure** **8**a,b). After *Vd* inoculation, the disease symptoms of TRV: *GbNFYA5* plants did not change significantly compared to TRV: 00 and Hai7124 plants (Figure 8c,d), However, the percentage of wilted leaves in TRV: *GhNFYA5* plants was significantly higher than that in TRV: 00 and TM‐1 plants (Figure 8e,f). The stereomicroscope was used to visually observe the accumulation of invading *Vd* in vascular tissues, and *GhNFYA5*‐silenced plants were less affected than control plants (Figure 8g,h). We further found that compared to control plants, the transcripts of *GhOSM1* were increased approximately fourfold in TRV: *GhNFYA5* plants, while the expression of *GbOSM1* was not changed in TRV: *GbNFYA5* plants (Figure 8i,j). These results demonstrate that GhNFYA5 is a negative regulator of VW resistance, and that it represses the expression of *GhOSM1* through direct binding to CCAAT element in the promoter of *GhOSM1*.

Taken together, these findings suggest that NFYA5 binds to the CCAAT element in the *GhOSM1* promoter in TM‐1 and inhibits *GhOSM1* expression, while *GbOSM1* is highly expressed in Hai7124 due to the CCAAT mutated into the CCGAT, and NFYA5 binding efficiency in the element is greatly decreased. The natural A/G variation in the *OSM1* promoter among different cotton accessions significantly affects the expression levels of *OSM1* and VW resistance.

## 3 Discussion

### 3.1 A Newly Identified *GbOSM1* Plays a Crucial Role in Conferring VW Resistance

Osmotin was initially discovered in tobacco as a disease‐resistant protein linked to osmotic stress.^[^ ^25^ ^]^ Subsequently, it has been identified in many plants and reported to be involved in disease resistance or osmotic stress responses. In Arabidopsis, *AtOsmotin34* was identified for the first time by screening the cDNA library using the osmotin gene in tobacco as a reference sequence.^[^ ^39^ ^]^ Nevertheless, the functional mechanism of osmotin has not been well studied in the last 20 years. Although *GhOSM34* in *G. hirsutum* has been reported to be involved in cotton disease resistance,^[^ ^40^, ^41^ ^]^ the *OSM1* in this study is a newly identified osmotin gene in cotton, clustered in different phylogenetic groups with *GhOSM34* (Figure S3, Supporting Information). *OSM1* exhibited a high expression level in the disease‐resistant *G. barbadense* acc. Hai7124 roots and obvious up‐regulation when induced by *Vd*. In contrast, it was not expressed in the disease‐susceptible *G. hirsutum* acc. TM‐1 roots (Figure 1a,b; Figure S2, Supporting Information). Different from other osmotin genes in cotton, only *GbOSM1* showed this unique expression pattern (Figure S21, Supporting Information), implying its important role in disease resistance. Osmotin plays a vital role in defensing against the pathogen attacks.^[^ ^33^, ^42^ ^]^ The expression of the tobacco osmotin gene improves plant resistance to fungal pathogens, such as *Phytophthora infestans*, *Colletotrichum gloeosporioides*, and *Fusarium pallidoroseum*. Its heterologous expression in soybeans also confers resistance against a range of pathogens.^[^ ^43^, ^44^ ^]^ In this study, we demonstrate that silencing *GbOSM1* in Hai7124 increased plant susceptibility to VW, while overexpression of *GbOSM1* in *G. hirsutum* conferred enhanced tolerance (Figure 2). In addition, we found that several chromosome segment introgression lines carrying *GbOSM1* of *Gossypium barbadense* acc. Hai7124 in TM‐1 genetic background exhibited the higher VW resistance compared to the control (Figure S22, Supporting Information). Our results indicate that *OSM1* is an important candidate gene for improving the VW resistance in cotton.

### 3.2 GbOSM1 has Significant Antifungal Activity by Hydrolyzing Polysaccharides of the *Vd* Cell Wall

*V. dahliae* is a highly aggressive pathogen, producing melanized dormant structures known as microsclerotia, which enable its survival in soil for several years.^[^ ^45^ ^]^ Upon infection, the germ tube of the microsclerotia emerges from the surface of the infected root, and extends longitudinally along the root epidermal cells. Subsequently, a few swollen hyphae form a narrow perforation peg that perforates at the junction of root epidermal cells, and penetrates the epidermis under pressure.^[^ ^46^ ^]^ The septin‐ring‐organized hyphal neck serves as a functional fungus‐host penetration interface for the delivery and secretion of toxins or secretory proteins.^[^ ^47^ ^]^ The association of fungal hyphae with the plant's woody vascular tissues, which are not easily accessible to fungicides. The mycelium blocking the xylem vessel, impairing the transport of water and nutrients within the plant. Toxins, such as acidic protein‐lipopolysaccharide complexes or secreted proteins, can severely disrupt the plant's metabolic processes or immune responses.^[^ ^48^, ^49^ ^]^ In response to *Vd*, plants have developed a wide array of defense mechanisms. In addition to constitutive physical and chemical barriers, plants also induce the expression of defense‐related proteins in reaction to the virulence of *Vd*, including several proteins initially classified as pathogenesis‐related (PR) proteins, such as osmotin.

Osmotin can accumulate in response to biotic stress and exhibits antifungal activity in plants. In vitro expression of one osmotin gene from *Vitis vinifera* inhibited *Botrytis cinerea* and *Phomopsis viticola* growth.^[^ ^50^ ^]^ Recombinant osmotin from tobacco exhibited significant antifungal activity.^[^ ^51^ ^]^ In the study, GbOSM1 also exhibited significant antifungal activity in vitro, including the inhibition of spore germination and hyphal growth of *Vd* (Figure 3a–g). It has been reported that several osmotin proteins with antifungal activity possess β‐1,3‐glucanase activity, although there is no conclusive evidence linking these two activities.^[^ ^52^ ^]^ Additionally, chitinases are another type of PR protein produced by plant cells in response to pathogen infection, capable of hydrolyzing chitin in fungal cell walls.^[^ ^21^ ^]^ Through the analysis of protein hydrolase activity, we discovered that the GbOSM1 primarily disrupts cells by breaking down the cell walls of fungi, including chitin and glucan (Figure 3h; Figure S12, Supporting Information). Within the TLP family, members of the acidic subclass are usually found in the extracellular space, while the basic subclass is typically located in the plant cell vacuole.^[^ ^53^ ^]^ Although osmotin are basic proteins, they contain a conserved acidic cleft domain (REDDD), which is believed to be an essential functional domain that binds to the fungal cell membrane and disrupts the plasma membrane.^[^ ^29^ ^]^ Our results revealed that GbOSM1 was stored in tonoplasts under normal conditions, but it was released into the apoplast to function under osmotic stress or stress stimulation by *Vd* (Figure 4). An acidic cleft domain, composed of REffDsDyD, was also predicted in the GbOSM1 protein, which may be critical for disrupting the β‐1,3/1,4/1,6 bonds in *Vd* cell wall polysaccharides and leading to cell death. (Figure 1d; Figure S5, Supporting Information). In addition, it has been reported that osmotin can induce apoptosis in fungi by binding to a receptor‐like polypeptide (PHO36).^[^ ^54^ ^]^ Thus, we imply that after disrupting the cell wall, GbOSM1 may affect cellular metabolism and induce apoptosis by binding to specific plasma membrane receptors in *Vd*, which remains to be further clarified.

### 3.3 Overexpression of *GbOSM1* Activates a Comprehensive Immune Response in Plants

It was reported that osmotin weakens pathogen defense and enhances plant virulence against pathogen cells by affecting the intracellular signal transduction pathway in pathogen, such as promoting the activation of the mitogen‐activated protein kinase (MAPK) cascade and the accumulation of ROS,^[^ ^31^, ^55^ ^]^ implying its potential role as a cell signal pathway modulator. Furthermore, osmotin regulates immune activation in plants, and it may act by binding to some cell surface receptor.^[^ ^56^ ^]^ In this study, we found that the GbOSM1 did not affect gene transcription levels of *Vd* based on RNA‐seq analysis (Figure S11, Supporting Information). However, overexpression of *GbOSM1* activated a comprehensive immune response in plants. GbOSM1 induced a local PCD response in tobacco, leading to the up‐regulation of the expression levels of abundant resistance related genes (Figure S17, Supporting Information). RNA‐seq and RT‐qPCR results showed that *GbOSM1* overexpression transgenic lines had high resistance to pathogens, and immune‐related signaling pathways were activated (Figure 5). In addition, the immune pathway was significantly inhibited after *Vd* infection in *GbOSM1*‐silenced plants (Figure S16, Supporting Information).

We suggest that *GbOSM1* is upregulated under stress conditions, and released into the plasma membrane and recognized by some pattern recognition receptors (PRRs), thus activating plant immunity. We found that the GbOSM1‐induced programmed cell death in *N. benthamiana* cells was more similar with that caused by the GbCERK1 (PRR), rather than the severe cell death induced by BAX (Apoptosis‐promoting Bcl‐2 Associated X Protein) and VdNLP1 (elicitor necrosis and ethylene‐induced protein) (Figure S17b–d, Supporting Information). In both cotton and *N. benthamiana*, this immune response might be highly comprehensive. For instance, cytoplasmic kinases such as GhBIK1, GhMAPK3, NbBAK1, and NbSOBIR1 transmit PRRs‐recognized signals to downstream, LOX1 regulates hormone signaling pathways, RBOHD affects the accumulation of ROS, calmodulin proteins modulate calcium ion signaling pathways, and transcription factors like WRKY and ERF regulate the expression of downstream defense genes such as PR and NBS‐LRR (Figure 5h; Figure S17f, Supporting Information). This forms a complex and comprehensive immune signaling network initiated by GbOSM1, protecting plants from external stresses.

### 3.4 Finetune Regulating the Expression of OSM1 Achieves the Trade‐Offs Between Plant Growth and Immunity

Plants have an array of defenses against pathogens, but initiation of any defense response often results in an unintended decrease in growth and yield. Exploring the regulatory mechanism between plant immunity and growth, and finetune regulating the expression of target genes within an optimal range is of great significance for the trade‐offs between plant growth and immunity.^[^ ^57^, ^58^ ^]^ The *OsOSM1* mRNA level in rice was positively correlated with the resistance of rice to sheath blight disease. Extremely high *OsOSM1* mRNA levels were detrimental to rice development, but appropriately increased mRNA levels could improve disease resistance while ensuring development and yield.^[^ ^32^ ^]^ Our results suggest that high mRNA levels of *GbOSM1* overexpression line (OE26) had no impact on cotton yield, while they did have partially adverse effects on cotton growth and fiber quality. Nevertheless, the moderate *GbOSM1* expression levels in OE28, OE32, and OE389 lines showed the minimal negative impact on plant growth and development (Figure S23, Supporting Information). Also, four Hai7124‐TM‐1 chromosome segment introgression lines carrying *GbOSM1* showed that there was no obvious difference in yield and fiber quality traits compared to TM‐1 (Table S4, Supporting Information). Thus, increasing the mRNA level of *GbOSM1* within a relatively appropriate content can enhance the VW resistance of cotton without causing adverse effects on agronomical traits, which can be applied in cotton disease resistance breeding.

### 3.5 Natural SNP Variation in the OSM1 Promoter Contributes to Cotton VW Resistance

In this study, we identified an A/G natural SNP variation in the *OSM1* promoter that contributed to differential expression of *OSM1* and VW resistance in cotton (Figure 6). The A/G SNP led to two haplotypes of CCAAT/CCGAT elements in the *OSM1* promoter of different cotton accessions. In animals and plants, NF‐YB and NF‐YC assemble as a heterodimer in the cytoplasm and subsequently translocate into the nucleus to interact with NF‐YA.^[^ ^38^ ^]^ The activated NF‐YA directly binds to the CCAAT element in the promoters of target genes and regulates gene transcription levels positively or negatively in a highly controlled manner.^[^ ^59^, ^60^ ^]^ There are 10 NFYA genes in Arabidopsis,^[^ ^61^ ^]^ but they exhibit low homology to the NFYA genes in cotton (Figure S19, Supporting Information). We identified an *NFYA5* gene in cotton that was significantly up‐regulated for expression upon induction by *Vd* (Figure S20a,b, Supporting Information). NFYA5 is a nuclear‐localized transcription factor with a conserved CCAAT binding domain despite extremely low homology among different plants (Figure S20c,d, Supporting Information). This suggests that NFYA5 is required to bind CCAAT and regulate downstream gene expression, regardless of its role in the plant life cycle. We have accumulated several lines of evidence supporting a repressive role of NFYA5 on *OSM1* expression in TM‐1. This repression is contingent upon the CCAAT/CCGAT natural variation present in the susceptible and resistant cotton plants. First, yeast one‐hybrid assays and EMSA experiments showed that NFYA5 bound to the CCAAT element but not the CCGAT element in the *OSM1* promoter (Figure 7a,b). Second, promoter‐regulated LUC and GUS reporter gene expression assays and enzyme activity analyses demonstrated that NFYA5 repressed *OSM1* promoter activity in TM‐1 (Figure 7c–f). Third, when *GhNFYA5* was silenced in TM‐1, the expression of *GhOSM1* significantly increased, leading to enhanced plant disease resistance. In contrast, silencing of *GbNFYA5* had no effect on Hai7124 (Figure 8). Thus, NFYA5 negatively regulates OSM1 expression and VW resistance.

*G. barbadense* generally exhibits superior VW resistance compared to *G. hirsutum*.^[^ ^4^, ^11^ ^]^ However, the molecular mechanisms underlying differences in disease resistance remain unclear. To date, several reports have identified potential candidate resistance genes through genome‐wide association studies and differential transcriptome analyses in cotton. These DEGs induced by *Vd* infection are involved in the metabolism of lignin and jasmonic acid, and cell wall modification in various VW‐resistant lines.^[^ ^62^, ^63^, ^64^, ^65^ ^]^ Our population genetics study revealed that the *OSM1* promoter in the 335 *G. hirsutum* accessions had only the CCAAT haplotype. In contrast, the CCAAT/CCGAT haplotypes were present in the 269 *G. barbadense* accessions (Figure 6c,d). The expression of *OSM1* was higher in *G. barbadense* carrying the CCGAT haplotype, and the plants exhibited increased resistance to VW (Figure 6e–h). The recent report showed that the set of *G. barbadense* accessions were clustered into three groups, G1 and G2 mainly included modern cultivars from Xinjiang, China, and G3 was related to widely introduced accessions in different regions worldwide.^[^ ^66^ ^]^ We found that there was no distinct distribution difference of the two haplotypes in G3 group, with 58.88% CCGAT haplotype and 41.12% CCAAT haplotype, while 92.50% CCGAT haplotype in G2 group and 31.70% in G1 group (Table S5, Supporting Information). In addition, G1 had the better fiber quality compared to G2 group. Association analysis further showed that there was no obvious difference of fiber yield traits between the two haplotypes, while fiber uniformity, fiber elongation, and fiber strength were significantly lower in accessions carrying CCGAT haplotype (163 accessions) compared to those carrying CCAAT haplotype (106 accessions) (Figure S24, Supporting Information), which is consistent with our results that high mRNA levels of *GbOSM1* transgenic line OE26 has partially adverse effects on plant growth and fiber quality (Figure S23, Supporting Information). These findings indicated that modern *G. barbadense* cultivars in Xinjiang, China might undergo different breeding selection in balancing the fiber quality traits and resistance.

**Figure 9:**
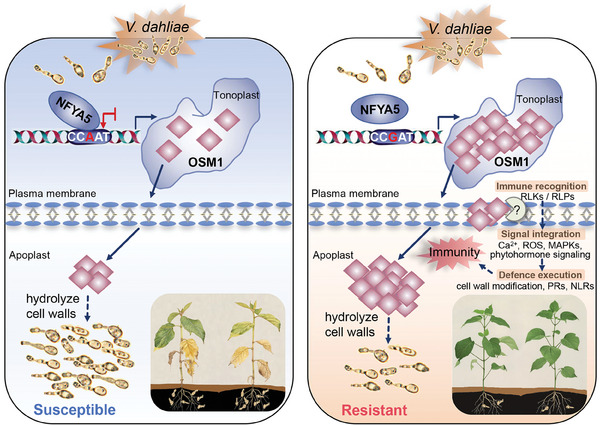
Schematic model of natural SNP variations in the *OSM1* promoter contributes to the differential transcription of *OSM1* and VW resistance. In all *G. hirsutum* and a few *G. barbadense* accessions, transcription factor NFYA5 directly binds to CCAAT element to repress the *OSM1* gene expression, further compromise the resistance to VW. In contrast, in most of *G. barbadense* accessions, NFYA5 was unable to bind to the CCGAT element in the promotor of *GbOSM1* due to A/G SNP variation, thus exhibited the high expression of *GbOSM1* and conferred the strong resistance to VW. GbOSM1 localizes to the tonoplast and is secreted extracellularly when *Vd* infection. Overexpression of *GbOSM1* prevented *Vd* infection through directly hydrolyzing pathogen cell wall polysaccharides and activating the plant immunity.

Based on these results, we propose a model in which the natural A/G variation in the *OSM1* promoter confers the differential transcription of *OSM1* and plant disease resistance (**Figure** **9**). *GbOSM1* positively regulates defense against *Vd* by hydrolyzing cell wall polysaccharides of *Vd* and activating the plant immune pathways. In natural populations, *G. hirsutum* GhNFYA5 binds to the CCAAT element and represses downstream *GhOSM1* gene expression, further compromises the resistance to VW. In contrast, there are A/G SNP variation in the *GbOSM1* promotor in *G. barbadense* accessions. The mutation of the A/G base results in that GbNFYA5 can not bind to the CCGAT element to suppress the expression of *GbOSM1*, leading to high transcripts level of *GbOSM1*, and exhibiting high VW resistance. As is well‐known, *G. hirsutum* has high yield and wide adaptability, but are susceptible to *Vd*. Thus, it is promising to enhance the VW resistance of *G. hirsutum* with various strategies by overexpressing *GbOSM1* by transgenic technology, crossing with chromosome segment introgression lines carrying CCGAT‐genotype *GbOSM1*, and genome editing A/G variation in the promotor of *GhOSM1*, with *G. hirsutum* target accession as improved receptor in the future. In addition, repressing *NFYA5* transcription also represents an effective strategy to enhance VW resistance of *G. hirsutum* accessions.

## 4 Experimental Section

### Plant Materials and Treatments

Wild‐type cotton (*G. hirsutum* acc. TM‐1 or cv. Junmian 1, and *G. barbadense* acc. Hai7124 showing susceptibility and resistance to *Vd*, respectively) and transgenic *GbOSM1* plants generated from *G. hirsutum* acc. W0 as receptor, were planted in the cotton experimental base of Nanjing Agricultural University in Dangtu, Anhui, China (118°E, 32°N) and Ledong, Hainan, China (108°E, 18°N), for investigation and propagation. Seedlings were grown in the same controlled environment chamber under the following conditions: 16 h light (28 °C)/8 h dark (25 °C) cycle and 60% humidity.

### Vd Strain Culture and Inoculation Treatments

The *Vd* V991 strain, a highly aggressive and defoliating strain of *Vd* obtained from the lab, was cultured on Potato Dextrose Agar (PDA) medium for 4–5 days. Subsequently, it was transferred to Czapek‐Dox liquid (CPK) medium for further incubation for five days (25 °C). The concentration of conidia was adjusted with sterile water to 1 × 10^7^ conidia mL^−1^, and the dip‐inoculation method was used to inoculate plant seedlings.^[^ ^67^, ^68^ ^]^ To detect the transcript levels of genes in TM‐1 and Hai7124 after V991 treatment, the roots of cotton seedlings were harvested at 0, 6, 12, 24, 48, and 72 h after inoculation, with three biological repeats for each treatment, respectively. RNA from root tissue was extracted for transcriptome sequencing and RT‐qPCR analysis.

### Plasmid Construction and Genetic Transformation

The open reading frame (ORF) of *GbOSM1* was fused into the pBI121 vector at the *Bam*H I and *Sac* I restriction sites to generate the *GbOSM1* overexpression vector. The plasmids were then introduced into *A. tumefaciens* (strain LBA4404). The *A. tumefaciens*‐mediated cotton transformation was carried out following a previously described method.^[^ ^69^ ^]^ Briefly, cottonseeds of *G. hirsutum* acc. W0, a high‐regeneration line selected from *G. hirsutum* cv. Jinmian25 in the laboratory, were surface‐sterilized with 30% (v/v) hydrogen peroxide. After rinsing with sterile distilled water, the seeds were germinated on solidified 1/2 MS medium to obtain hypocotyls. After 7 days of growth, the hypocotyls were excised into 5–6 mm segments and co‐cultivated with *A. tumefaciens* LBA4404 cells for 8–10 min. Subsequently, the hypocotyl segments were cultured on a medium containing kanamycin. Calli and embryogenic calli were induced from the ends of the hypocotyl segments. After 8–10 months, putative regenerated transgenic plants were obtained. These plants were then planted and self‐pollinated. The homozygosity of the transgenic plants was determined by PCR‐based genotyping for the presence or absence of the transgene, along with segregation analyses of the kanamycin selection marker. The primers used are listed in Table S6 (Supporting Information).

### RNA Isolation and RT‐qPCR Analysis

For gene expression analysis, the total RNA was extracted using the RNA Extraction Kits (CW2598S, CWBIO Co., Ltd, Beijing, China), and the RNA samples were reverse‐transcribed into cDNA using the HiScript III RT SuperMix for qPCR (+gDNA wiper) (R323, Vazyme Inc., Nanjing, China). The gene‐specific primers for RT‐qPCR analysis were designed using Beacon Designer 7.0. Cotton histone 3 (AF024716) was used as a reference gene. All reactions were conducted in triplicate, and Primers used are listed in Table S6 (Supporting Information). All RT‐qPCRs were performed on a Bio‐Rad CFX‐96 PCR system using ChamQ SYBR qPCR Master Mix (Q311, Vazyme Inc. Nanjing, China).

### VIGS Experiments

The *GbOSM1* gene‐specific fragment was inserted into the TRV2 vector. The empty vectors TRV: 00 and TRV: *GbCLA1* (cloroplastos alterados1) were used as negative and positive controls, respectively. The plasmids were transformed into *A. tumefaciens* strain GV3101 and injected into the cotyledons of cotton seedlings.^[^ ^70^ ^]^ Two weeks after injection, the RNAs were extracted from roots to detect the transcripts level of *GbOSM1* using RT‐qPCR analysis. At least 30 plants of each VIGS treatment were selected for the V991 infection assay, and the ratio of diseased to healthy leaves was investigated. To analyze invasive growth in cotton, the stems infected with *Vd* were cut and observed using a stereoscope (LEICA DVM6, Germany). Primers used are listed in Table S6 (Supporting Information).

### Fungal Biomass Assays

To quantify the relative biomass of V991 infiltrating into the plant interior, stems were collected for DNA extraction after ten days of treatment with V991. The ribosomal DNA's Internal Transcribed Spacer (ITS) region was targeted using the *Vd*‐specific ITS1‐F primer and STVe1‐R reverse primer. Primer for histone 3 in cotton was used as an endogenous plant control. The qPCR was performed on genomic DNA.^[^ ^71^ ^]^ Primers used are listed in Table S6 (Supporting Information).

### Determination of the Antifungal Activity of GbOSM1 In Vitro

For the expression and purification of the GbOSM1 protein, the *GbOSM1* (without SP) was inserted into the pCold TF DNA vector with a protein tagged (6 × His) to construct His‐fusion plasmid. The *E. coli* strains BL21 (DE3) were individually transformed with His and His‐GbOSM1 fusion protein, inducing the expression of recombinant proteins using 0.8 mM IPTG at 25 °C overnight. The proteins were purified using Ni‐NTA Agarose Resin (L00250, Genscript, Nanjing, China). The purity of the proteins was determined by resolving them on a 10% (w/v) SDS‐PAGE gel. Then, the His‐GbOSM1 protein was detected by immunoblotting using an anti‐His antibody (A02003, Genscript, Nanjing, China).

The antifungal activity of the purified protein against *Vd* was assessed using the disc agar diffusion method. The 100 µL V991 spore suspensions (1  ×  10^4^ conidia mL^−1^) were initially evenly coated on PDA medium for one day at 25 °C. Afterward, three sterile Whatman filter paper discs with a diameter of 1.5 cm were placed onto plates. Different concentrations of His‐GbOSM1 purified protein were added to the Whatman filter paper discs. His purified protein and ddH_2_O were added to the discs as negative controls. Plates were incubated at 25 °C for three days and then photographed.

To observe and measure the growth of the hyphal. The *Vd* were incubated on the PDA medium at 25 °C for 7 days and then transferred to 24‐well cell culture plates (84013ES03, Yeasen Biotechnology Co., Ltd., Shanghai, China) containing 4 × concentrated Potato Dextrose Broth (200 µL) and 0, 50, 100, or 200 µg mL^−1^ of His‐GbOSM1 purified protein. The spore suspensions were incubated at 25 °C for 4 days and photographed.^[^ ^72^ ^]^ The concentration of the medium under each treatment condition was measured, and the inhibition rate of mycelial growth was calculated. The concentration was represented by the OD_600_ value. Inhibition rate was calculated using the formula: Inhibition rate = ［OD_600_ (0 µg mL^−1^) – OD_600_ (50, 100, or 200 µg mL^−1^)］/ OD_600_ (0 µg mL^−1^) × 100%.

For conidial production tests, the V991 spore suspensions (1  ×  10^4^ conidia mL^−1^) were incubated in Czapek‐Dox (CPK) liquid medium with varying concentrations of His‐GbOSM1 and His purified protein on a shaking table at 160 rpm at 25 °C for five days. The fungal cells were specifically stained with Periodic Acid‐Schiff (PAS), where a darker red color indicated a higher total number of conidia. The precise number of conidia was counted under a microscope using the hemocytometer counting method, and photographs were captured with a light microscope (Olympus BX53, Tokyo, Japan) after the spore suspension was diluted tenfold.

### Enzyme Activity Assays

To extract the components of the fungal cell wall, the fungus was first cultured on PDA medium and then transferred to CPK medium for five days (25 °C, 160 rpm). Subsequently, the culture was filtered through gauze to collect the mycelium. The mycelium was ground and transferred to the cell wall crushing solution (50 mM Tris‐HCl, 5 mM EDTA, and 1 mM PMSF), then the cell wall was crushed using a high‐pressure cell crusher (JN‐mini, JNBIO, Guangzhou). A 12 000 rpm centrifugation for 30 minutes was used to collect the mycelium fragments, which were then washed with sterile water and lyophilized. After adding a buffer (50 mM Tris‐HCl, 5 mM EDTA, 40 mM β‐Mercaptoethanol, and 2% SDS) to the lyophilized samples, they were heated at 100 °C for 10 min, and then washed with sterilized water to eliminate any remaining protein residues. Finally, the lipids were removed using an extraction with chloroform‐methanol‐water (1:1:0.3) solution for 12 h. The isolated cell wall components were collected, rinsed with sterile water and lyophilized for processing.^[^ ^73^ ^]^

The amount of reducing sugars released from fungal cell walls, such as Glucan, Chitin, and other related polysaccharides by GbOSM1 was analyzed using the 3,5‐dinitrosalicylic acid (DNS) method. His protein and boiled His‐GbOSM1 were used as the negative controls, respectively. The standard reaction system included 10 µg of GbOSM1 recombinant protein dissolved in PBS buffer and 0.5% (w/v) of polysaccharide‐related substrates dissolved in 200 µL of sodium acetate (100 mM, pH 5.2). The entire reaction system was first incubated at 50 °C for 15 min. Subsequently, 400 µL of DNS reagent was added and the mixture was further incubated at 100 °C for 10 min. After cooling, the optical density was measured at 540 nm using a spectrophotometer (Bio‐Rad Laboratories, Inc., CA, USA). Enzymatic activity was designated as micrograms of glucose produced per minutes per milligram of protein. Data were collected from four biological replicates in each assay.

### Subcellular Localization of GbOSM1

To observe the localization of GbOSM1 in plant protoplasts, the ORF of *GbOSM1* was fused to EGFP. Protoplast extraction was performed using the plant protoplast preparation and transformation kit (RTU4072, Real‐Times Biotechnology Co., Ltd, Beijing, China) following manufacturer's instructions. Briefly, four‐week‐old Arabidopsis leaves were cut into thin strips with a blade and submerged in an enzymatic hydrolysate solution (provided in the kit) at room temperature for 3–4 h. The protoplasts were then washed with solution 2 and re‐suspended in solution 3 (provided in the kit). The fusion constructs of GbOSM1‐EGFP were transformed into Arabidopsis protoplasts using the polyethylene glycol‐induced method. After incubating the transformed protoplasts at 28 °C in the dark for 12–16 h, GFP and chloroplast auto‐fluorescence signals were observed using a confocal microscope (SP8, Leica, TCS, Germany). Excitation wavelengths and emission filters were adjusted to 488 nm/band‐pass 490–550 nm for GFP, and 488 nm/band‐pass 650–720 nm for chloroplast auto‐fluorescence.

To observe the localization of GbOSM1 in *N. benthamiana* leaf cells, the ORF of *GbOSM1* was fused to GFP in the pBinGFP4 expression vector. The vector was transiently expressed in *N. benthamiana* leaf cells using the *A. tumefaciens* infiltration method, and co‐expressed in leaves with tonoplast, cell membrane, or peroxisomal marker genes.^[^ ^74^ ^]^ Three days after infiltration, the fluorescence signals were detected using a confocal microscope (LSM780, Zeiss, Jena, Germany) at 488 nm and 561 nm, respectively.

### Programmed Cell Death Analysis

For the observation of localized cell necrosis in *N. benthamiana* leaves, vectors carrying different genes were transiently expressed in *N. benthamiana* leaf cells using the *A. tumefaciens* infiltration method as described above. *A. tumefaciens* was injected in a volume of 200 µL. The necrotic phenotype of the leaves was observed and photographed after 4–5 days.

For Trypan Blue staining, the *N. benthamiana* leaves were placed flat in the container and immersed in Plant Trypan Blue Stain Solution (G4808, Solarbio Life Science, Beijing, China) for staining about 2–3 h. After that, the leaves were removed and washed off the excess dye with 95% ethanol, then immersed in 95% ethanol and sealed for decoloration at least 12 h. The leaves were observed and photographed after soaking in distilled water for 30 min.

For Electrolyte leakage assay, the cell death induced by proteins was analyzed by measuring ion leakage from leaf discs as described.^[^ ^75^ ^]^ Eight leaf discs (d = 0.8 mm) from agriculturally infiltrated leaves were collected and floated with shaking at 50 rpm in 4 mL of sterile water for 3 h. Ion leakage was quantified by analyzing the conductivity of water before and after boiling the sample.

### Transcriptome Sequencing

The plant tissue samples and *Vd* were collected after treatment with V991 or H_2_O, respectively. Total RNA was extracted and used to construct the RNA‐seq libraries. Sequencing was performed on an Illumina NovaSeq6000 platform. After removing the adapters with Cutadapt, the reads were mapped to *G. hirsutum* acc. TM‐1 (NAU_v2.1) genome using a Hisat2 with default parameters.^[^ ^76^ ^]^ The number of matched reads was determined using HTSeq software and then imported into R statistical software for DEG analysis with DESeq2 (*q‐value* < 0.05, fold change > 2).^[^ ^77^ ^]^ The gene expression values were normalized using FPKM analysis. Hierarchical clustering analysis, GO, and KEGG analysis were performed using the Omicshare website (https://www.omicsshare.com/).

### Yeast One‐Hybrid (Y1H) Assay

The Matchmaker Gold Yeast One‐Hybrid System (Clontech) was used to investigate whether the GhNFYA5 protein could bind to the CCAAT/CCGAT motif. The CCAAT/CCGAT/CCCCT elements were cloned into the yeast expression vector pHis to construct the pHis‐bait plasmids, respectively. The *GhNFYA5* gene was cloned into the pGADT7 vector to generate pGADT7‐GhNFYA5. The polyethylene glycol‐mediated method co‐transformed the *S. cerevisiae* Y1H Gold strain with pHis‐ and pGADT7‐based vectors. The DNA‐protein interactions were assessed based on the growth status of yeast cells cultured on the SD/‐Trp/‐Leu/‐His selective medium with 40 mM 3‐AT (the minimal inhibitory concentration) for three days at 30 °C.

### Electrophoretic Mobility Shift Assay (EMSA)

The 55‐bp promoter fragments of *GbOSM1*/*GhOSM1* containing the CCGAT, CCAAT, and mutant CCCCT elements were labeled with biotin and used as the detection probe. The unlabeled competitor DNA probe and mutated DNA probe were synthesized. *GhNFYA5* was constructed in the pGEX‐4T vector and induced with 0.8 mM IPTG at 25 °C for 12 h. The labeled probe signals were detected using the Light Shift Chemiluminescent EMSA kit (RE231894, Thermo Fisher Scientific, Waltham, MA, USA). Specifically, the purified GhNFYA5 protein and DNA probe were incubated for 30 minutes at room temperature in EMSA/Gel‐Shift binding buffer (GS005, Beyotime Biotechnology, Shanghai, China). The DNA‐protein complexes were separated on a 6.5% polyacrylamide gel, transferred to a nylon membrane (INYC00010, Biosharp Life Sciences, Hefei, China), and detected according to the manufacturer's instructions.

### Promoter‐LUC Activity Assay

For the LUC assay, 1371‐bp *GbOSM1*/*GhOSM1* promoter fragments containing the CCAAT or CCGAT element (*pGhOSM1* and *pGbOSM1*) was amplified with specific primers and fused upstream of mini35S‐LUC to form the reporter vector pGreen II 0800‐LUC. The constructed pBI121‐35S::*GhNFYA5/GbNFYA5* vector and the empty pBI121 vector were used as the effector and negative control, respectively. The effector and reporter plasmids were transformed into *A. tumefaciens* strain GV3101 cells. *A. tumefaciens*‐mediated transformation of tobacco leaf cells was conducted using mixtures of *A. tumefaciens* cells harboring the effector and the reporter at a 5:1 ratio. The infiltrated tobacco plants were incubated in the dark for 12 h and then transferred to normal growth conditions for 48 h at 25 °C. The LUC activities were detected using a histochemical method and captured with a low‐light cooled charge‐coupled device camera (Tanon 5200 Multi, Shanghai, China).

The luciferase (LUC) and renilla luciferase (REN) activities were determined using the dual‐Luciferase Reporter Assay System (Promega, Madison, USA) with an Infinite200 Proreader (Tecan, Switzerland). The transient expression assay was performed with three biological replicates. The promoter activity was expressed as the ratio of LUC/REN.

### GUS Activity Determination

For the GUS assay, the 1371 bp promoter fragment, containing the CCAAT/CCGAT motif was cloned into the pBI121 vector, replacing the 35S promoter, which was used as the reporter. Then, pBI121‐35S::*GhNFYA5/GbNFYA5* vectors were constructed and used as effectors. An empty vector (unmodified pBI121 vector) was used as a control. *A. tumefaciens*‐mediated transformation of tobacco leaf cells was performed as described in the LUC assay. The infiltrated tobacco plants were incubated in the dark for 12 h and then transferred to normal growth conditions for 48 h at 25 °C. The GUS activities were detected and photographed using the histochemical method described previously.^[^ ^78^ ^]^ Primers used are listed in Table S6 (Supporting Information).

### Phenotypic Analysis of Cotton Yield Components and Fiber Quality Traits

For yield components, including boll number, boll weight, lint percentage, and seed index. At the mature stage of cotton (about 70% boll open), ten plants from each accession within each plot were randomly chosen from the middle of each row. The boll number was recorded using ten biological replicates for each accession. The boll weight was measured using twenty mature bolls randomly harvested. After ginning, the lint percentage was calculated as the ratio of lint weight to seed‐cotton weight, and the seed index was determined by weighing 100 properly developed seeds. Each line was represented by three biological replicates for the analysis.

For fiber quality traits analysis, mature fibers (25 g per sample) were collected from similar middle fruit branches at the same time for quality testing. The parameters measured included Fiber Length, Strength, Uniformity, and Micronaire, using a high‐volume fiber testing system (Premier HFT 9000, Premier Evolvics Pvt Ltd, Coimbatore, India). The evaluation of fiber quality parameters was conducted by the Center of Cotton Fiber Quality Inspection and Testing, Ministry of Agriculture and Rural Affairs (Anyang, Henan Province, China). Each accession was represented by three biological replicates for the analysis.

### Statistical Analysis

At least three independent biological replicates for each experiment were used in this study. The figure legends provide detailed information on sample size (n) for each statistical analysis. The standard deviation (SD) of the mean can be visualized in the figures as error bars. Stst 1.0 software was used to conduct statistical analysis. To determine significant differences between the two groups, the two‐tailed paired Student's *t*‐test was used. Asterisks (*) indicate statistical significance at *P* < 0.05, double asterisks (**) and letters (A/B/C) indicate statistical significance at *P* < 0.01.

## Conflict of Interest

The authors declare no conflict of interest.

## Author Contributions

W. G., X.W., and G.W. designed the research. G.W., J.K., Z.C., C.R., C.D., Z.G., H.L., Q.Z., and H.W. performed the research. G.W., D.Z., X.W., W.L., and W.G. analyzed the data. G.W., X.W., and W.G. wrote the paper. W.G. revised the paper.

## Supporting information



Supporting Information

Supplemental Movie 1

Supplemental Movie 2

Supplemental Tables S1‐S6

## Data Availability

The data that support the findings of this study are available from the corresponding author upon reasonable request.
