# Supplementary material for: Natural SNP Variation in GbOSM1 Promotor Enhances Verticillium Wilt Resistance in Cotton
Source: Adv Sci (Weinh). 2024 Oct 16;11(45):2406522. doi: 10.1002/advs.202406522 (PMC11615771; doi:10.1002/advs.202406522)
Supplement: Supplementary file 1 — Supporting Information [file ADVS-11-2406522-s004.pdf]

ADVANCED SCIENCE
Open Access

Supporting Information

for *Adv. Sci.*, DOI 10.1002/advs.202406522

Natural SNP Variation in *GbOSM1* Promotor Enhances Verticillium Wilt Resistance in Cotton

*Guilin Wang, Dayong Zhang, Haitang Wang, Jinmin Kong, Zhiguo Chen, Chaofeng Ruan, Chaoyang Deng, Qihang Zheng, Zhan Guo, Hanqiao Liu, Weixi Li, Xinyu Wang* and Wangzhen Guo**

**Supplemental information**

# Natural SNP Variation in *GbOSM1* Promotor Enhances Verticillium Wilt Resistance in Cotton

Guilin Wang, Dayong Zhang, Haitang Wang, Jinmin Kong, Zhiguo Chen, Chaofeng Ruan, Chaoyang Deng, Qihang Zheng, Zhan Guo, Hanqiao Liu, Weixi Li, Xinyu Wang *, Wangzhen Guo *

**Corresponding authors:**

Wangzhen Guo (moelab@njau.edu.cn);

Xinyu Wang (xywang@njau.edu.cn)

**This PDF file includes:**

Supplemental Figures S1 to S24

## Supplemental Figures

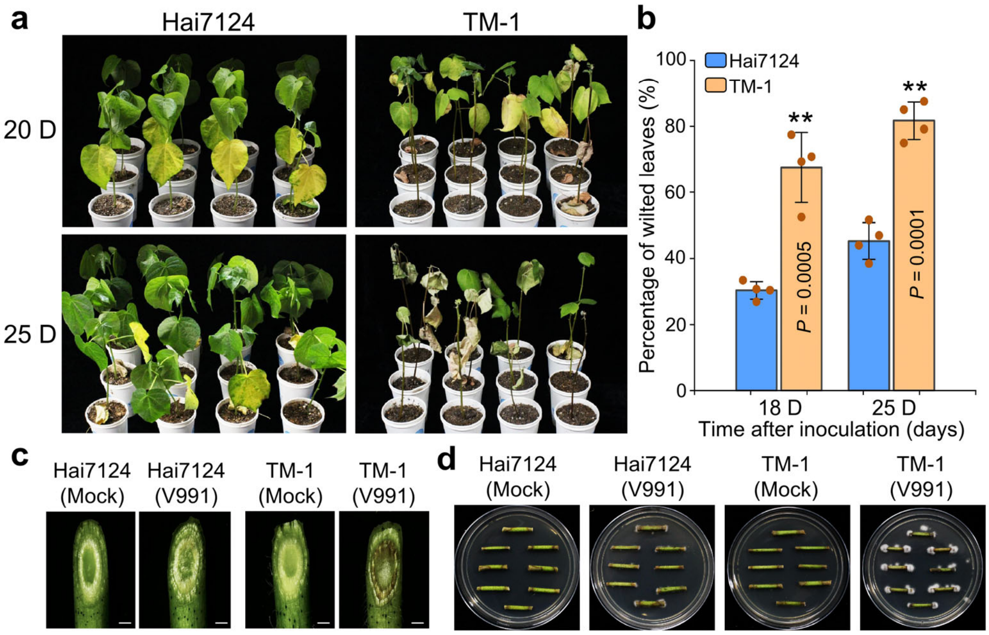


**Figure S1 Disease symptoms in *G. barbadense* acc. Hai7124 and *G. hirsutum* acc. TM-1. a)** Disease symptoms of Hai7124 and TM-1 plants were observed at 20 and 25 days after inoculation with *Vd*. **b)** Percentage of wilted leaves in Hai7124 and TM-1 plants after *Vd* inoculation. Each biological replicate contains at least 30 seedlings. Error bars represent the standard deviation of four biological replicates. Asterisks indicate statistically significant differences, as determined by the Student's *t*-test (**$P$<0.01). **c)** Vascular discoloration in Hai7124 and TM-1 plants after inoculation with *Vd*. Photographs were taken with a stereoscope (Olympus MVX10, Tokyo, Japan) for 10 days after inoculation. Scale bars: 1.5 mm. **d)** Fungal recovery experiments. Stem sections of Hai7124 and TM-1 plants at 10 days after inoculation were cut, placed on PDA plates and incubated at 25 °C. Photographs were taken three days after culture.

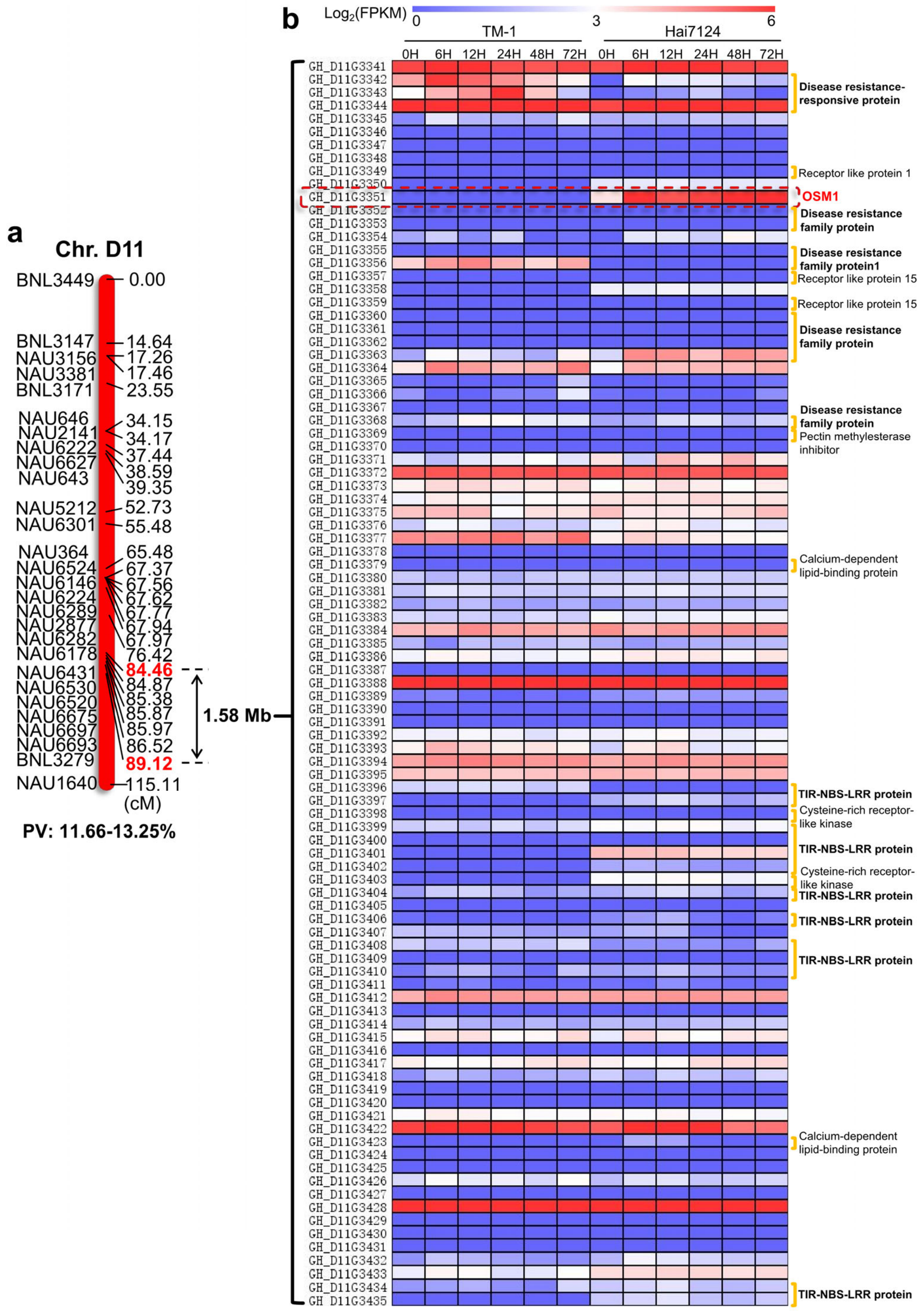


**Figure S2 Fine mapping of Verticillium wilt resistance-related QTL interval and candidate gene mining in Hai7124. a)** The resistance locus was delimited to a 1.58 Mb interval on Chromosome D11 with the total phenotypic variance (PV) ranging from 11.66-13.13% by using (Junmian1 × Hai7124) × Junmian1 $BC_1$ segregating populations. **b)** A total of 95 genes were predicted within the 1.58 Mb interval. The RNA-seq data showed that *GbOSM1* (marked as *OSM1* in red) expression and *Vd*-induced expression levels were higher in the disease-resistant cotton

Hai7124 roots than that in TM-1. A heat map was generated with Multi Experiment Viewer V.4.9 (http://en.bio-soft.net/chip/MeV.html). The expression data were converted to $log_2$ (FPKM) to calculate the expression level of genes. Colored squares indicated expression levels from 0 (blue) to 6 (red).

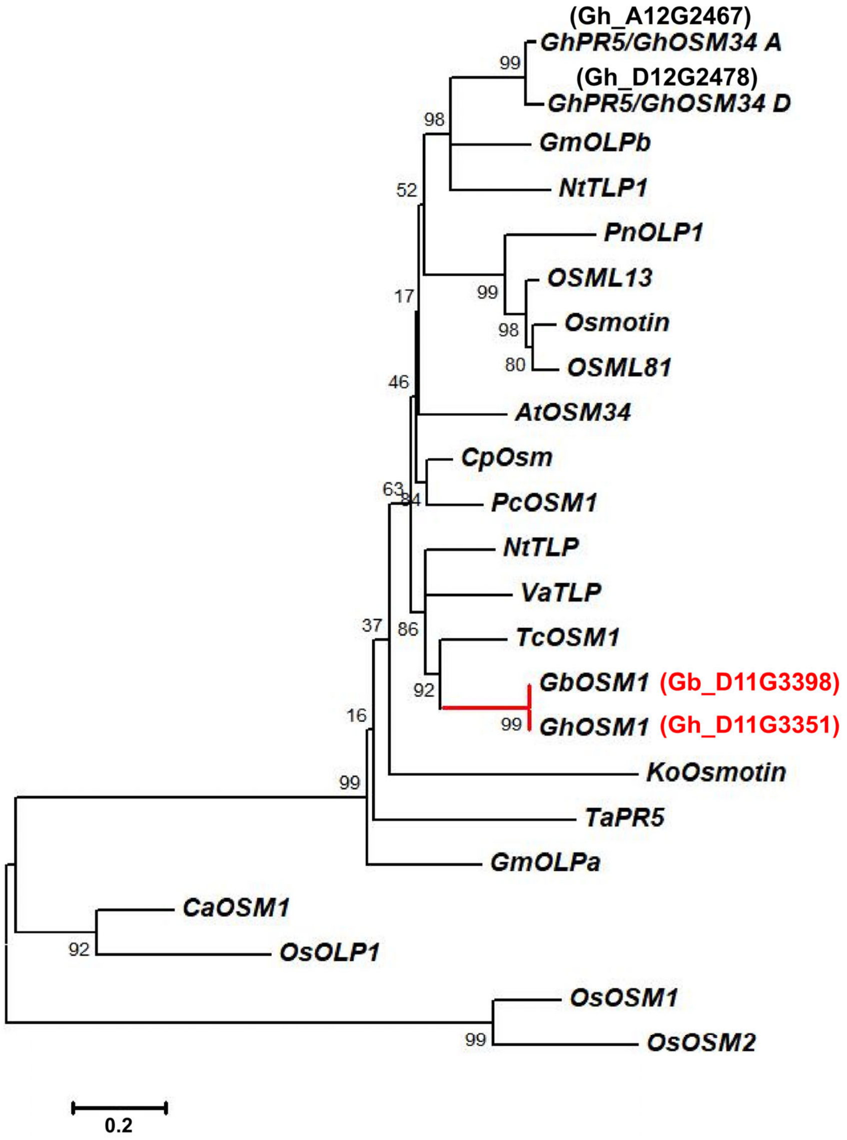


**Figure S3 Phylogenetic analysis of osmotin homologs reported previously in different plant species.** *GhPR5*/*GhOSM34* from *Gossypium hirsutum*, *GmOLPa* and *GmOLPb* from *Glycine max*, *NtTLP1*, *Osmotin* and *NtTLP* from *Nicotiana tabacum*, *PnOLP1* from *Panax notoginseng*, *OSML13* and *OSML81* from *Solanum commersonii*, *AtOSM34* from *Arabidopsis thaliana*, *CpOsm* from *Calotropis procera*, *PcOSM1* from *Pueraria colubrinum*, *VaTLP* from *Vitis amurensis*, *TcOSM1* from *Theobroma cacao*, *KoOsmotin* from *Kalanchoe obovata*, *TaPR5* from *Triticum aestivum*, *CaOSM1* from *Capsicum annuum*, *OsOLP*, *OsOSM1* and *OsOSM2* from *Oryza sativa*. The homology relationship between *GbOSM1*/*GhOSM1* and *TcOSM1* is the closest. Almost all of these proteins are involved in antifungal processes or in response to osmotic stress in plants.

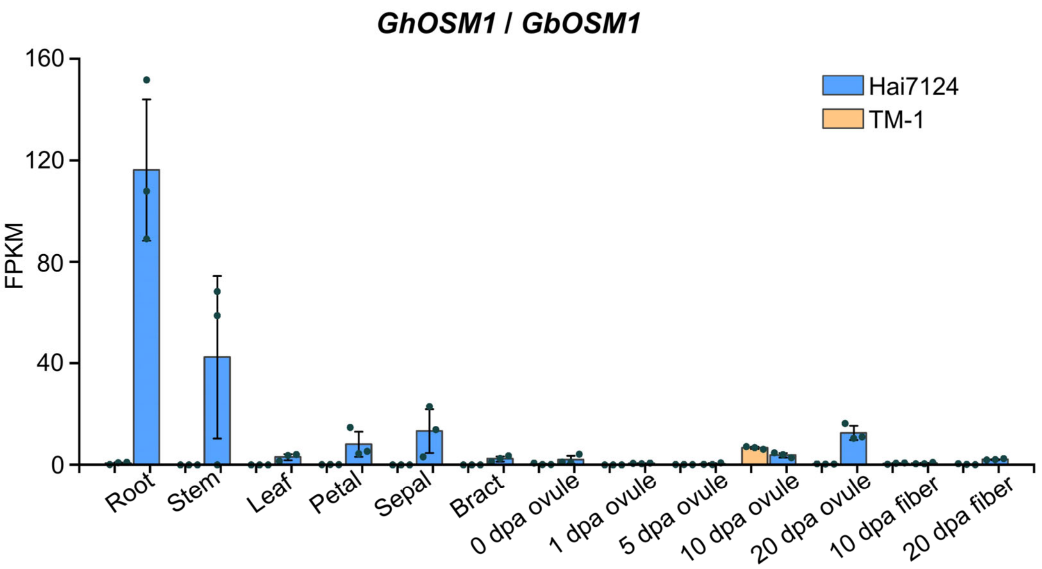


**Figure S4 Expression pattern of *GbOSM1*/*GhOSM1* in various tissues in *G. hirsutum* acc. TM-1 and *G. barbadense* acc. Hai7124.** The expression data were converted to FPKM to calculate the expression levels of *GbOSM1*/*GhOSM1*. Error bars represent the standard deviation of three biological replicates. The RNA-seq data were from http://www.ncbi.nlm.nih.gov/bioproject/ PRJNA248163/ and https://www.ncbi.nlm.nih.gov/bioproject/?term=G.+barbadense+and+Zhe jiang. Root, Stem, Leaf, Petal, Sepal, Bract, Ovules at 0, 1, 5, 10, 20 day post anthesis (dpa), and fibers at 10 and 20 dpa were used for expression analysis.

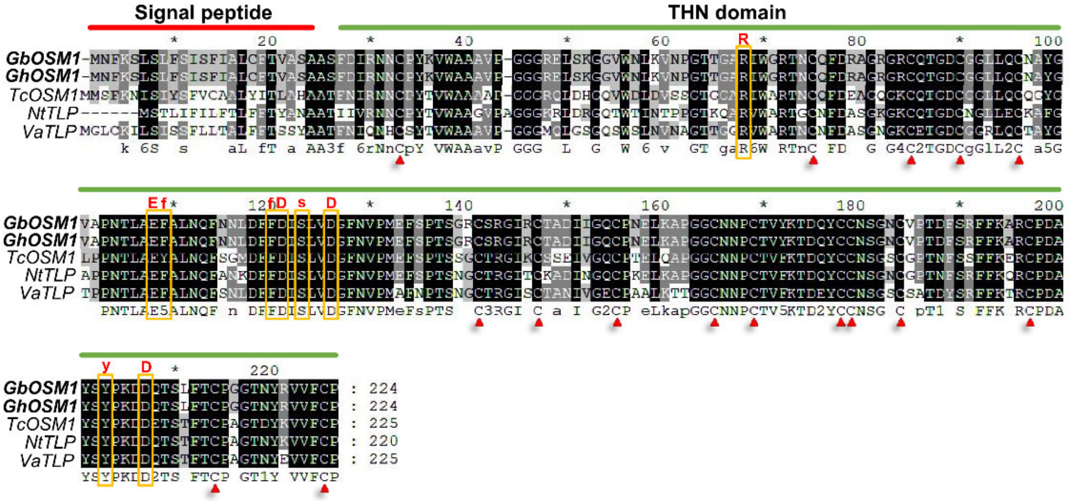


**Figure S5 Sequence alignment of OSM1 homologs from different plant species.** Amino acid sequence alignment of OSM1 homologs in *Gossypium hirsutum* (*Gh*), *Gossypium barbadense* (*Gb*), *Theobroma cacao* (*Tc*), *Vitis amurensis* (*Va*) and *Nicotiana tabacum* (*Nt*) was performed using GENEDOC. Identical amino acid residues are highlighted in black. The yellow boxes show an acidic cleft structure, REffDsDyD. The red arrows show 16 conserved cysteines.

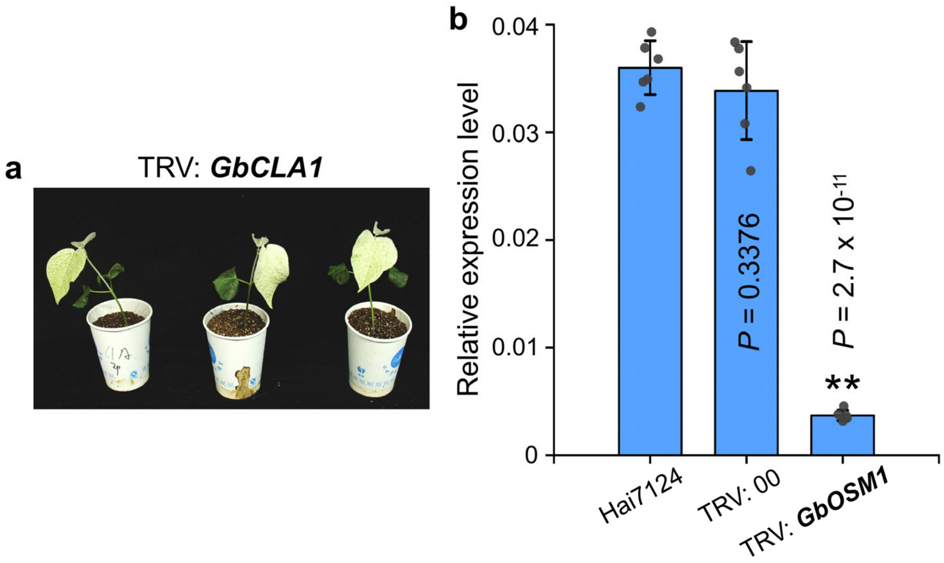


**Figure S6 Verification of VIGS silencing system. a)** Silencing of the endogenous cloroplastos alterados gene (*GbCLA1*) in cotton through tobacco rattle virus (TRV)-mediated virus-induced gene silencing (VIGS). Seven-day-old cotton seedlings (Hai7124) with two fully expanded cotyledons were infiltrated with TRV: *GbCLA1*, and the leaf bleaching phenotype was observed two weeks later. **b)** The efficiency of *GbOSM1* silencing was verified by RT-qPCR in VIGS plant leaves. Error bars represent the standard deviation of six biological replicates. Statistical analyses were performed using Student's *t*-test (***P*<0.01).

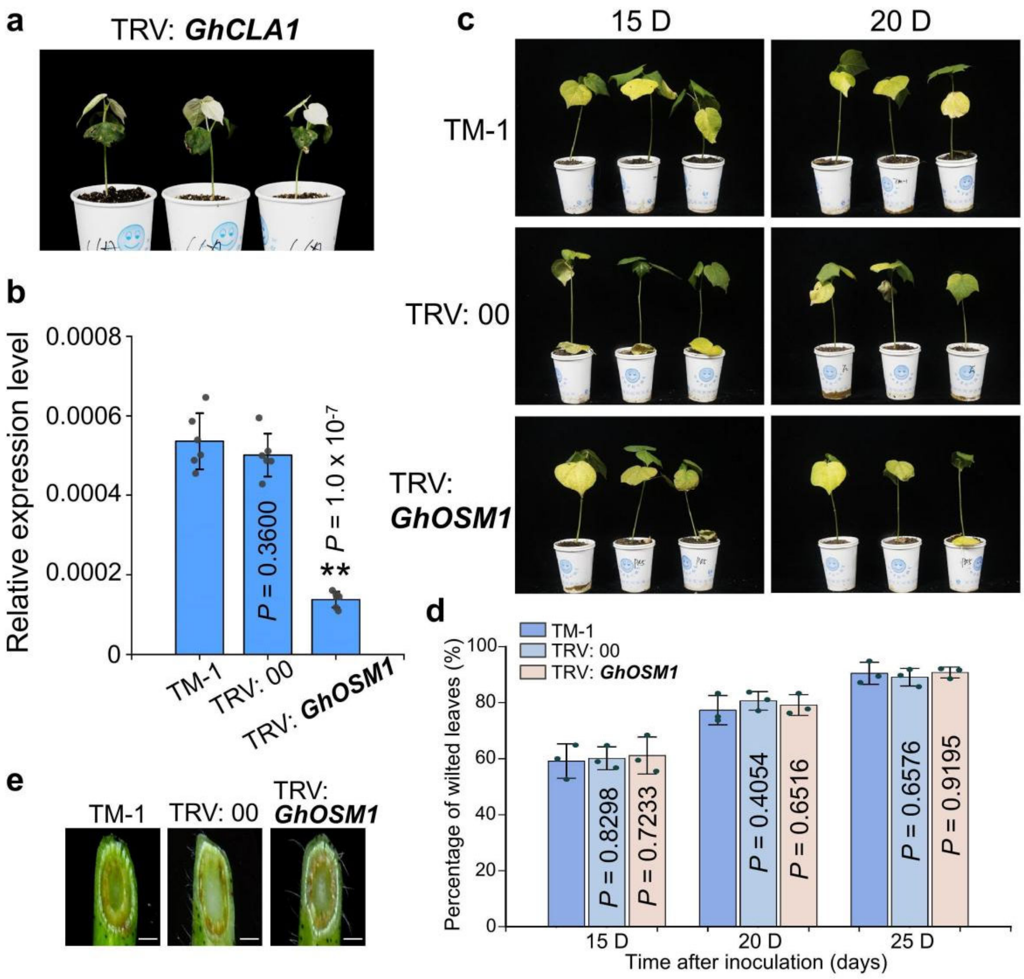


**Figure S7 Silencing *GhOSM1* in *G. hirsutum* acc. TM-1 does not observe obvious difference of disease resistance compared to TRV: 00 plants. a)** Silencing of the endogenous cloroplastos alterados gene (*GhCLA1*) in cotton through tobacco rattle virus (TRV)-mediated virus-induced gene silencing (VIGS). Seven-day-old cotton seedlings (TM-1) with two fully expanded cotyledons were infiltrated with TRV: *GhCLA1*, and the leaf bleaching phenotype was observed two weeks later. **b)** The efficiency of *GhOSM1* silencing was verified by RT-qPCR in VIGS plant leaves. Error bars represent the standard deviation of six biological replicates. Statistical analyses were performed using Student's *t*-test (**$P$<0.01). **c)** Disease symptoms of *GhOSM1*-silenced cotton plants were observed at 15 and 20 days after *Vd* inoculation. **d)** Percentage of wilted leaves in *GhOSM1*-silenced cotton plants after *Vd* inoculation. Each biological repeat contains at least 30 seedlings. Error bars represent the standard deviation of three biological replicates. Statistical analyses were performed by comparing with Hai7124 using Student's *t*-test. **e)** Vascular discoloration was observed in *GhOSM1*-silenced plants compared to the controls after inoculation

with *Vd*. Photographs were taken with a stereoscope (Olympus MVX10, Tokyo, Japan) at 10 days after inoculation. Scale bars: 1.5 mm.

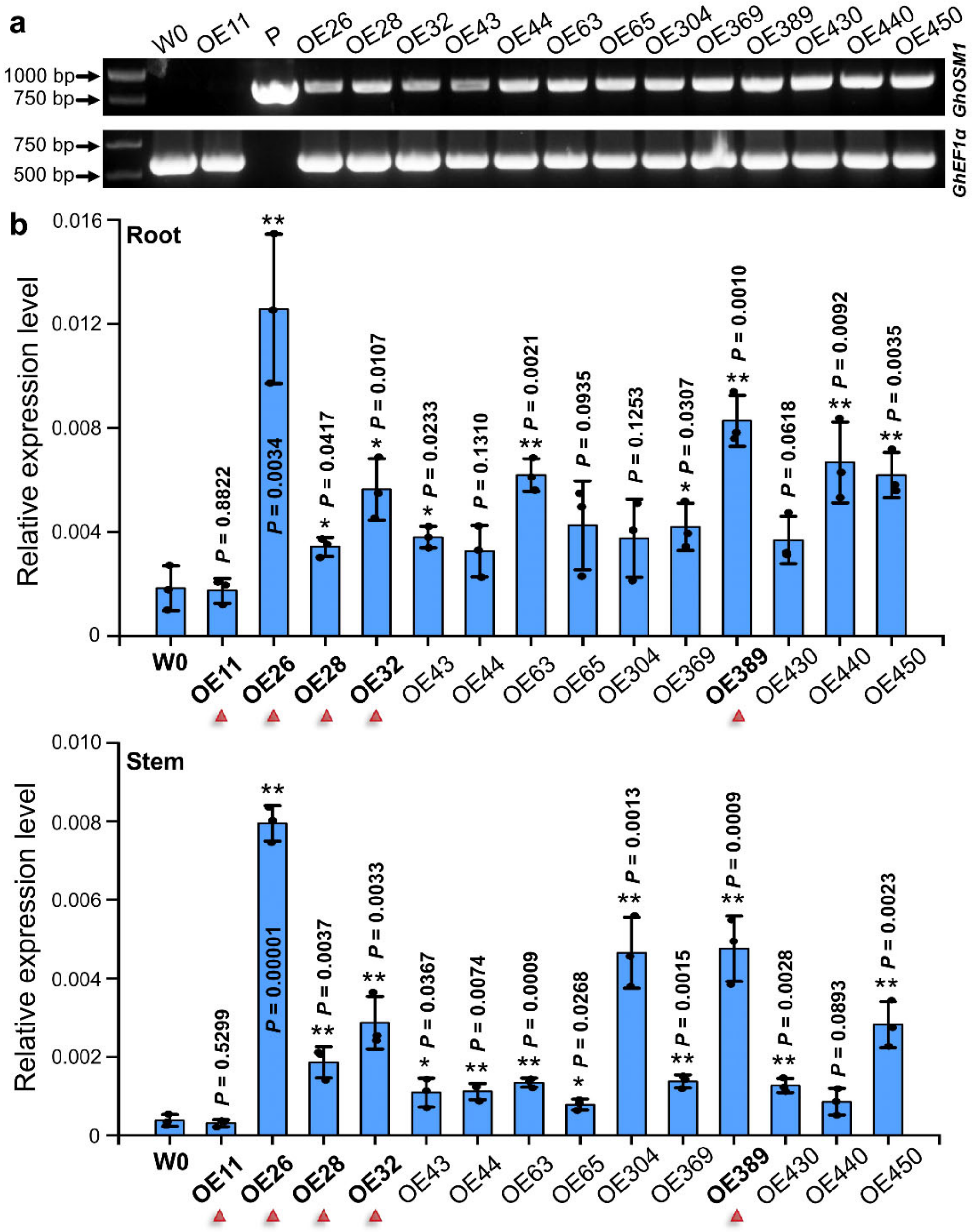


**Figure S8 Identification of overexpressing *GbOSM1* transgenic cotton lines. a)** *GbOSM1* was verified by PCR using genomic DNA in different cotton transgenic lines, with cotton *GhEF1α* (XM_041112012.1) as the internal control. **b)** *GbOSM1* transcripts level in root and stem of different transgenic lines was individually quantified by RT-qPCR using cotton *histone 3* (AF024716) as the internal control. W0, wildtype cotton; P, plasmid vector; OEs represent the different transgenic cotton lines, respectively. OE11, a negative control obtained during tissue culture. Error bars represent the standard deviation of three biological replicates. Statistical analyses were performed using Student's *t*-test (**P*<0.05, ***P*<0.01).

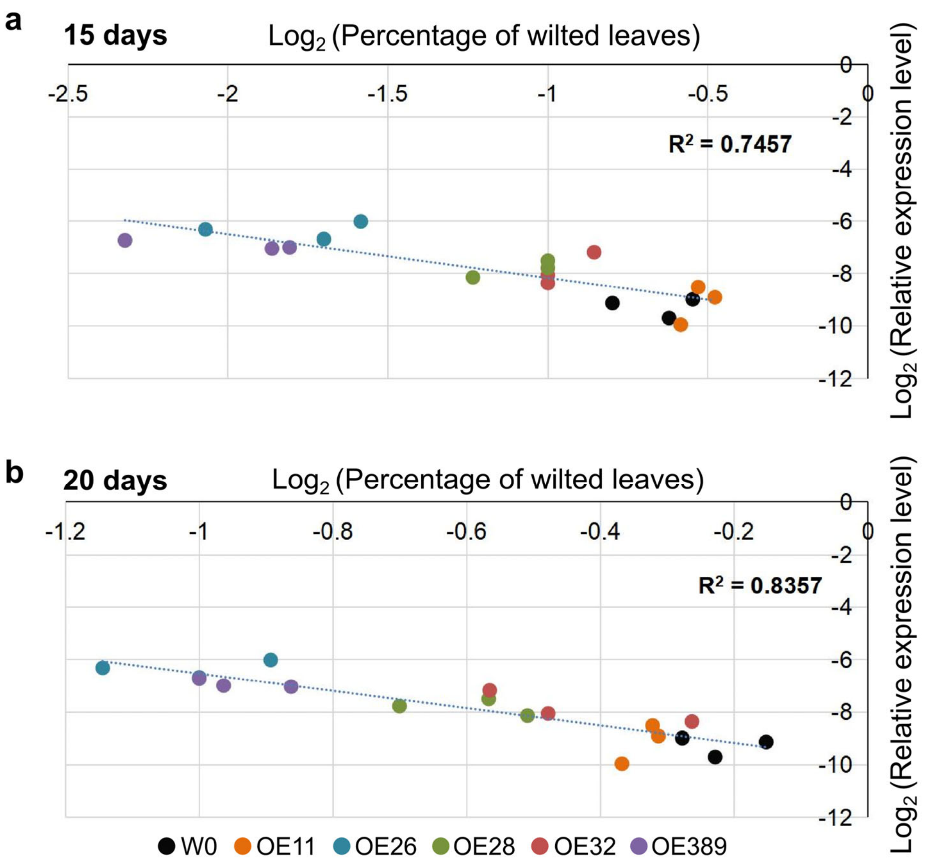


**Figure S9 Relationship between the relative expression level of *GbOSM1* and the percentage of wilted leaves in different transgenic cotton lines.** After 15 days **a)** and 20 days **b)** of *Vd* infection, the relative expression level $\log_2$ (expression ratio) values were plotted against the $\log_2$ (expression ratio) obtained by the percentage of wilted leaves. Standard curves were drawn, and the $R^2$ value was calculated to test the fitness.

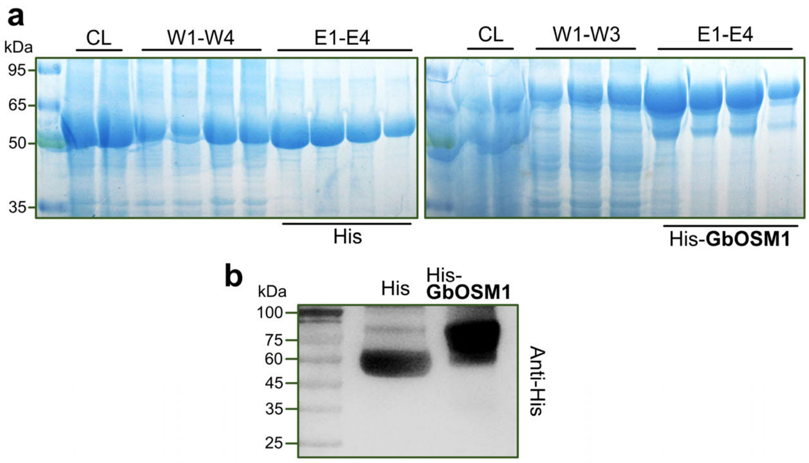


**Figure S10 Expression and purification of GbOSM1 protein *in vitro*. a)** Purification and SDS-PAGE analysis of recombinant His and His-GbOSM1 proteins expressed in the *E. coli* strain BL21 (DE3). Samples were resolved by 10% SDS-PAGE. CL, cell lysate. W, wash. E, elution. **b)** Immunodetection of His and His-GbOSM1 purified proteins using a His antibody. The molecular weight marker indicates the sizes of protein molecules.

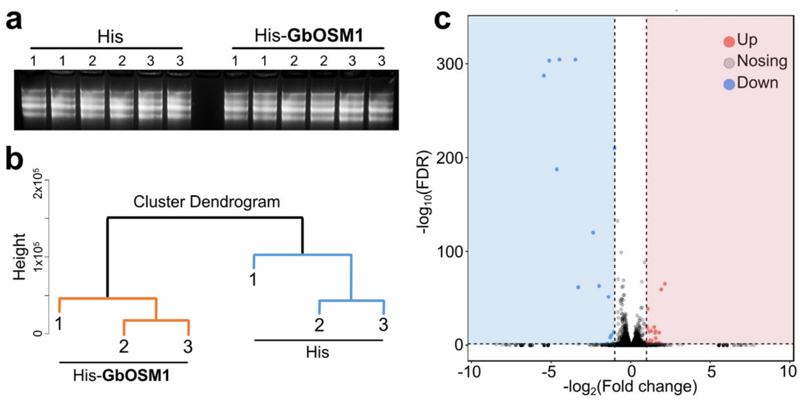


**Figure S11 RNA-seq analysis of *Vd* treated by purified GbOSM1 protein. a)** Total RNA was extracted from *Vd* spores' suspension treated for two days with His-GbOSM1 and His proteins, respectively. The RNA concentration was adjusted to be consistent, and transcriptome sequencing was performed. **b)** Clustering dendrogram of the six RNA-seq samples with three biological replicates for each treatment. **c)** Analysis of differentially expressed genes between two treatments.

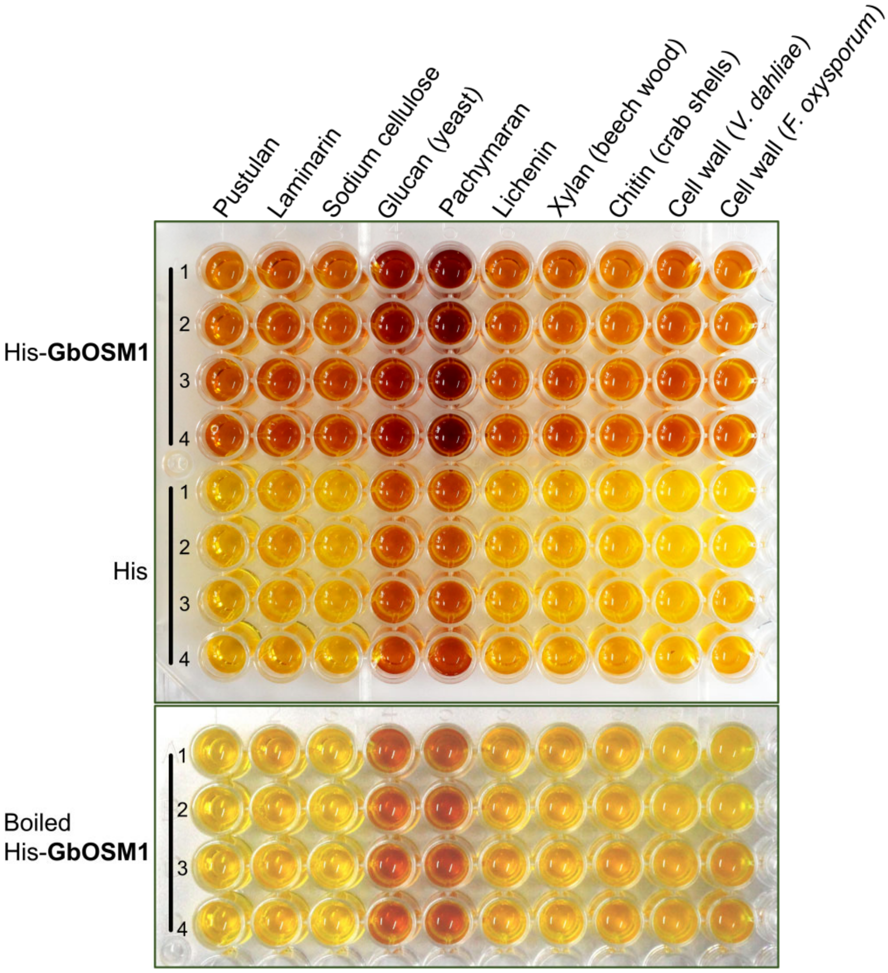


**Figure S12 GbOSM1 hydrolase activity assay.** The His-GbOSM1 hydrolyzes fungal cell walls and associated polysaccharides. A darker color indicates a higher content of reducing sugars released after the hydrolysis of polysaccharides. His protein and boiled His-GbOSM1 were used as the negative controls, respectively.

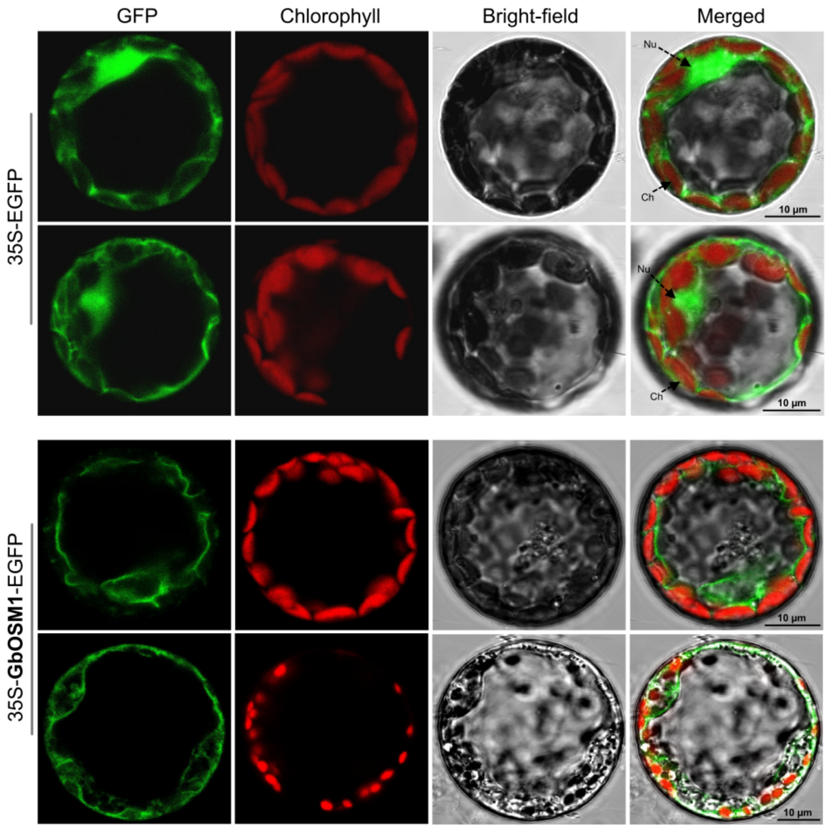


**Figure S13 Subcellular localization in Arabidopsis protoplasts shows that GbOSM1 is localized to the tonoplast.** Arabidopsis leaf protoplasts transiently expressing EGFP and GbOSM1-EGFP fusion protein were imaged by confocal microscopy (SP8, Leica, TCS, Germany). Fluorescence signals from GFP, chlorophyll autofluorescence, and the merged and bright-field images are shown. Nu, nuclear; Ch, chloroplast. Scale bars: 10 μm.

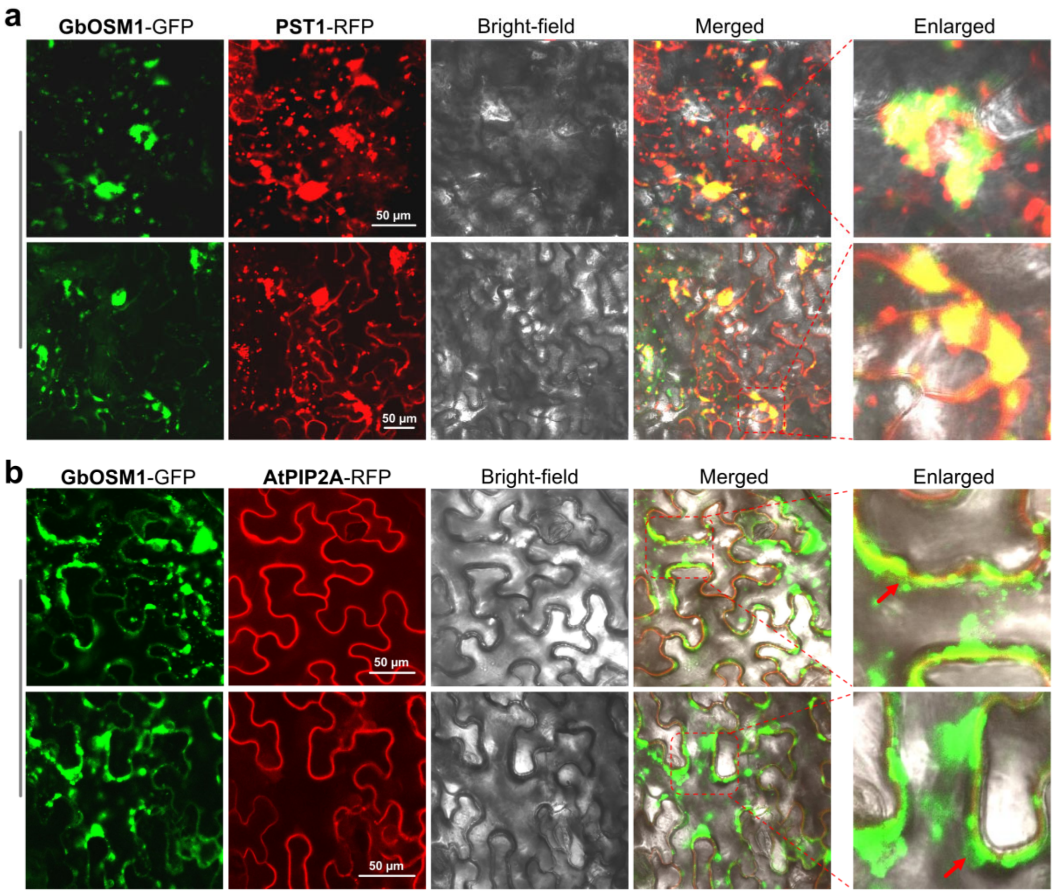


**Figure S14 GbOSM1-GFP is not localized to peroxisomes, but distributed near the plasma membrane in *N. benthamiana* leaf cells. a)** Co-localization analysis of GbOSM1-GFP fusion with a peroxisome marker, PTS1-RFP (red fluorescent protein carrying a typical peroxisome-targeting signal sequence PTS1, Ser-Lys-Leu). The enlarged portion shows peroxisomal red fluorescent signals localized near the vesicles, but not overlapping. **b)** GbOSM1-GFP fusion co-localizes with a plasma membrane marker, AtPIP2A-RFP. Red arrow indicates the distribution of GbOSM1-GFP green fluorescence close to the plasma membrane. Images were captured by confocal microscopy (LSM 780; Zeiss). Scale bars: 50 μm.

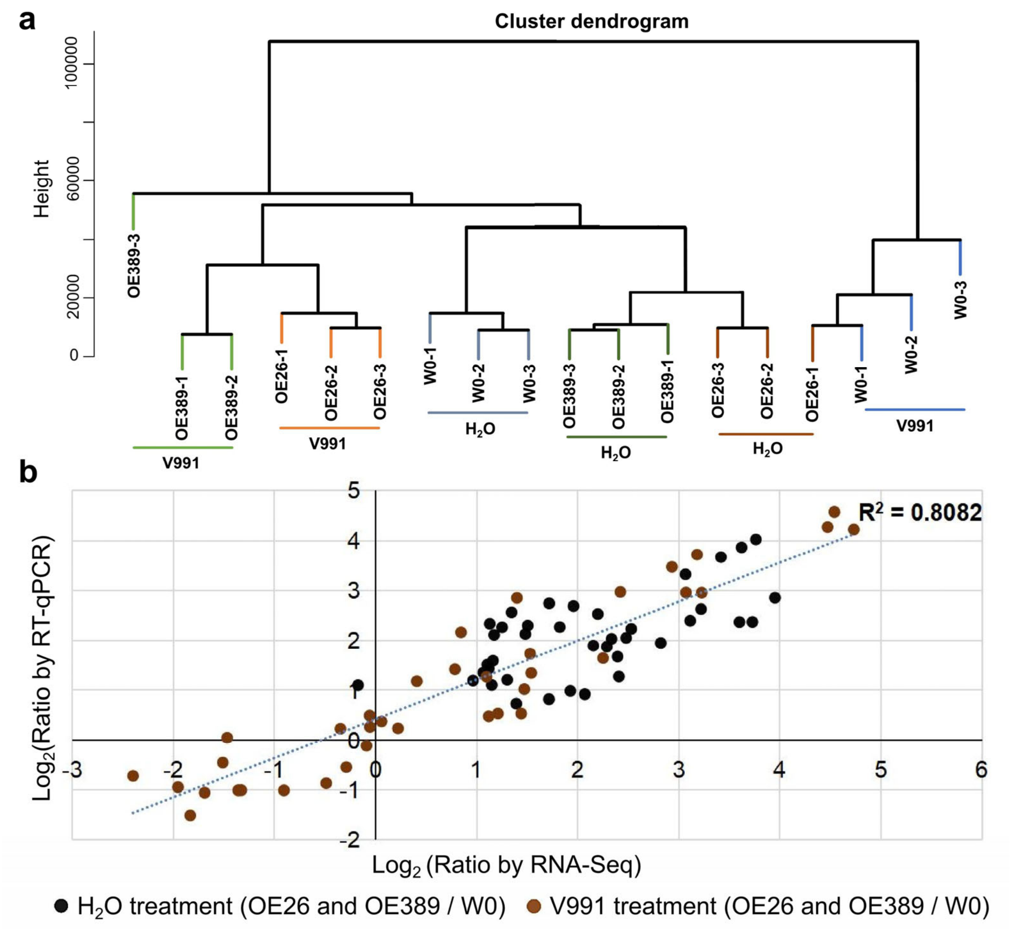


**Figure S15 RNA-seq assay in OE26 and OE389 transgenic cotton plants. a)** Clustering dendrogram of the 21 RNA-seq samples with three biological replicates for each treatment. After treatment with $H_2O$ and *Vd* for three days, the roots of each transgenic line and control were collected for RNA-seq analysis. **b)** Correlation of fold change analyzed by RNA-seq data with results obtained from RT-qPCR. Expression data were from 19 genes under two conditions ($H_2O$ and *Vd* treatments) for both WT and *GbOSM1* transgenic lines. The RNA-seq $\log_2$ (expression ratio) values were plotted against the $\log_2$ (expression ratio) obtained by RT-qPCR and standard curves. The $R^2$ value was calculated to assess the fitness of the data.

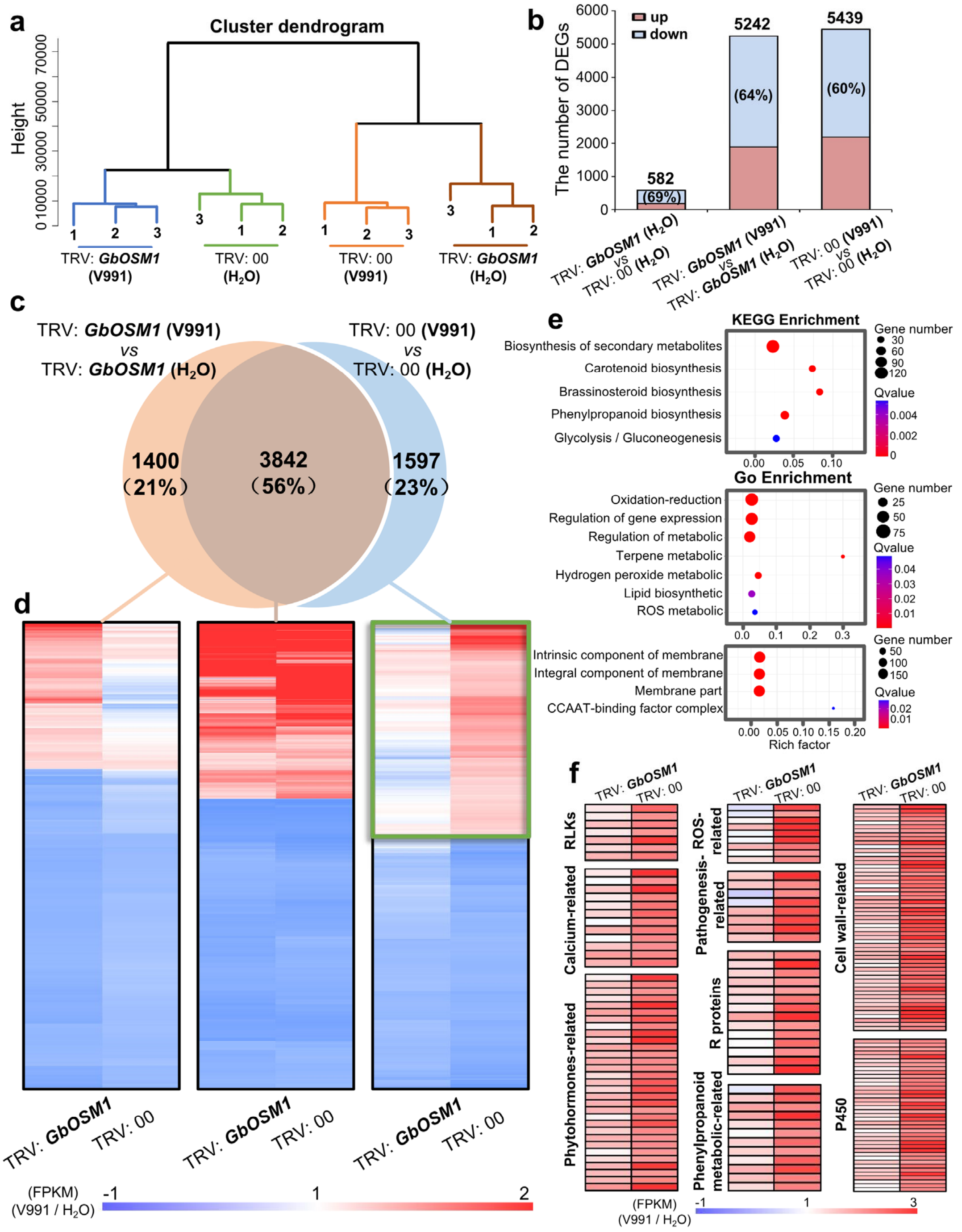


**Figure S16 RNA-seq assay of TRV: *GbOSM1* and TRV: 00 plants. a)** Clustering dendrogram of the 12 RNA-seq samples with three biological replicates for each treatment. Root samples of TRV: *GbOSM1* and TRV: 00 plants infected with *Vd* or treated with $H_2O$ for three days were collected for RNA-seq analysis, respectively. **b)** The number of DEGs in the roots of TRV: *GbOSM1* and TRV: 00 plants via different comparisons. The red represents the number of up-regulated DEGs, while the blue represents the number of down-regulated DEGs in the histogram. **c)** The number of

DEGs between TRV: *GbOSM1* or TRV: 00 plants after being challenged with *Vd* for three days. **d)** Expression clustering analysis of DEGs in the three parts mentioned in c). **e)** KEGG and GO enrichment analysis of DEGs in the green box in d). GO terms were separately shown by enrichment analysis. *P value* of 0.05 adjusted by false discovery rate (FDR). **f)** Heatmap of immune-related DEGs in the green box in d). The numerical values on the blue-to-red gradient bar represent the fold change-FPKM standardization values of the DEGs in each sample under *Vd* and $H_2O$ treatments.

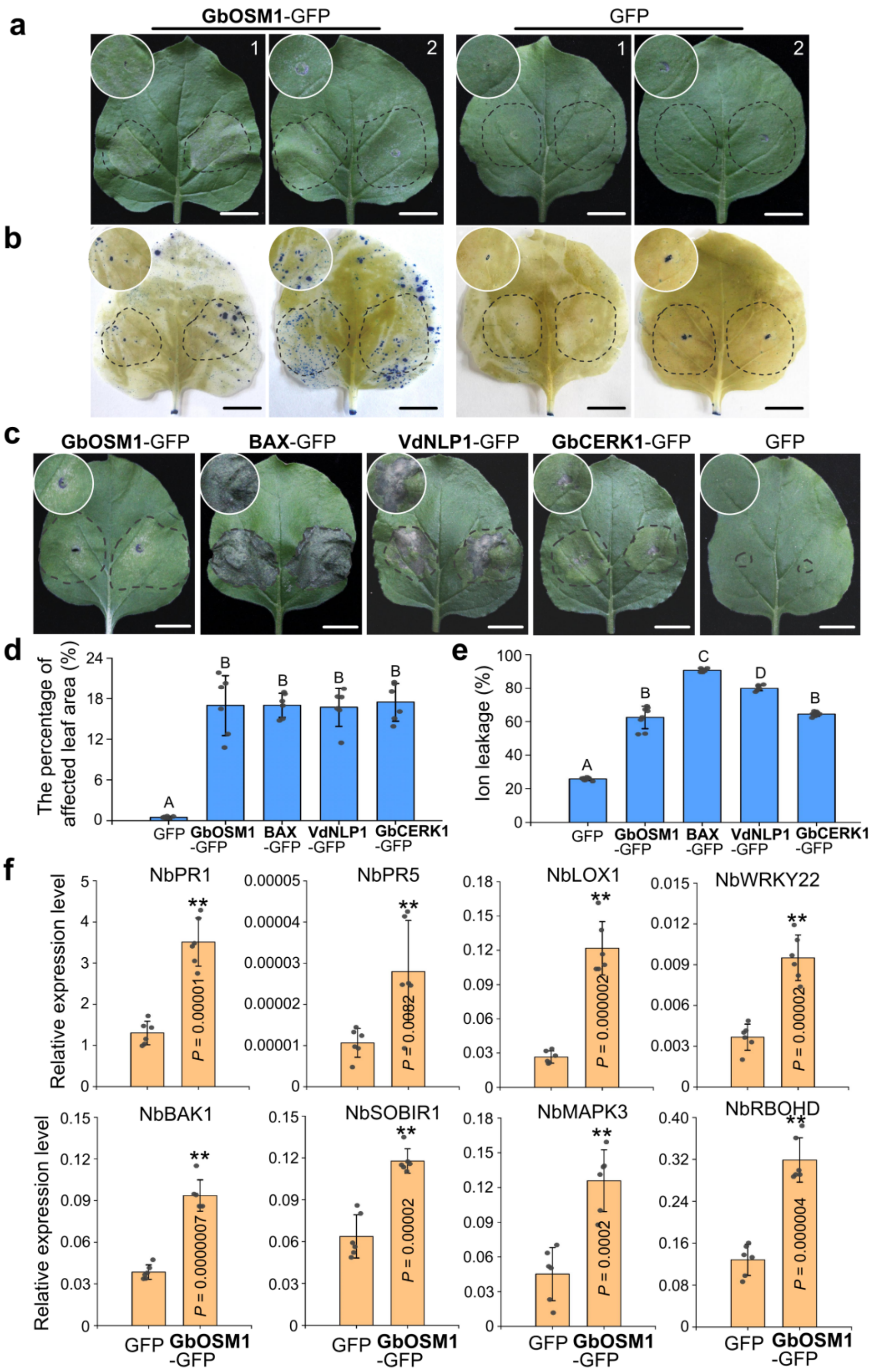


**Figure S17 GbOSM1-GFP activates the immune response when expressed in *N. benthamiana* leaves. a)** GbOSM1 caused programmed cell death in tobacco leaf cells. *Agrobacterium* harboring 35S: GbOSM1 construct was infiltrated into *N. benthamiana* leaves for transient expression with two biologic replicates for each construct. Scale bars: 1.0 cm. **b)** Trypan Blue staining for programmed cell death. **c)** Apoptosis-promoting Bcl-2 Associated X Protein (BAX, *NG_012191.1*), elicitor necrosis and ethylene-induced protein (VdNLP1, *VDAG_04701T0*),

pattern recognition receptor (GbCERK1, *GB_A11G1612*) were utilized as references to assess the level of programmed cell death. The local cellular immune response elicited by *GbOSM1* is more similar to that of *GbCERK1*. Scale bars: 1.0 cm. **d)** The percentage of necrotic area relative to the total leaf area in *N. benthamiana* leaves was analyzed using ImageJ software. Error bars represent the standard deviation of six biological replicates. Different letters indicate significant differences (one-way ANOVA; $P<0.01$). **e)** Quantification of cell death by electrolyte leakage measurement. Electrolyte leakage from infiltrated leaf discs was measured. Data are the means of eight independent experiments and error bars represent the standard deviation. Different letters indicate significant differences (one-way ANOVA; $P<0.01$). **f)** The expression of immune-related genes in *N. benthamiana* was validated by RT-qPCR. Error bars represent the standard deviation of six biological replicates. Statistical analyses were performed using Student's *t*-test (**$P<0.01$).

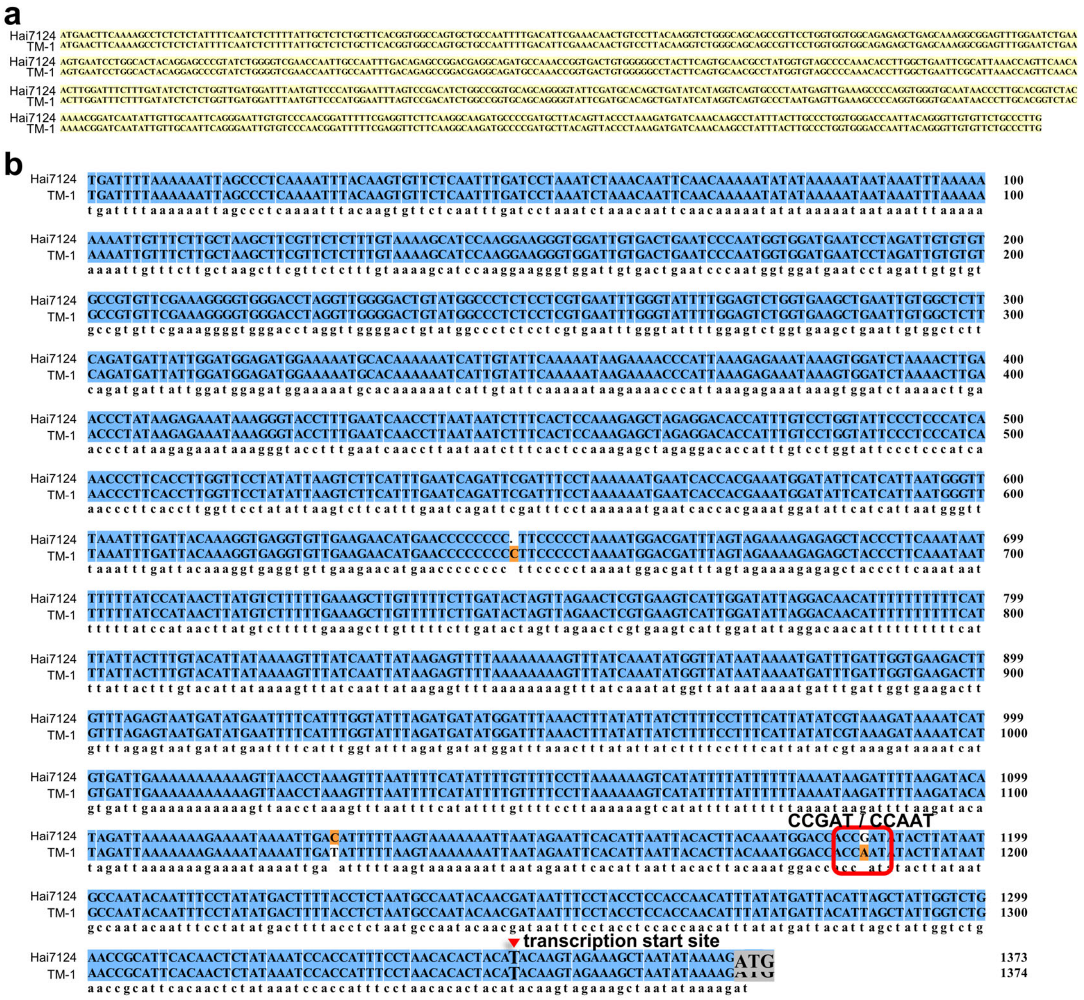

**Figure S18 Genome and promoter sequence analysis of *OSM1* homologs in Hai7124 and TM-1. a)** The open reading frame sequences of *OSM1* in Hai7124 and TM-1 were cloned separately and no base difference was detected. **b)** Cloning of *OSM1* promoter sequences in Hai7124 and TM-1. The red boxes show CCAAT/CCGAT element difference. The red arrow indicates the predicted transcription start site.

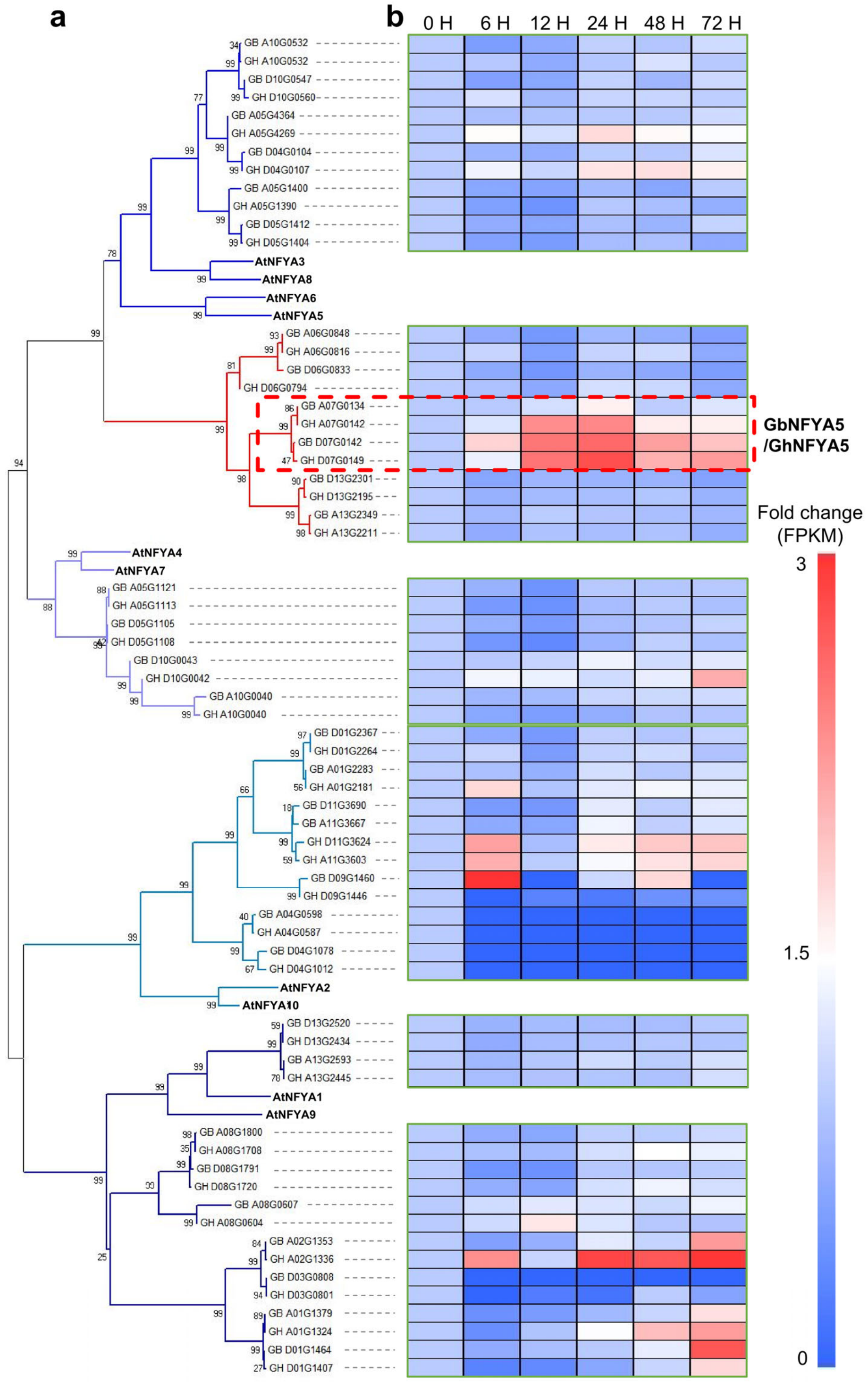


**Figure S19 Expression patterns of the NFYA family genes in response to *Vd* infection. a)** Phylogenetic relationship of NFYA family genes in Arabidopsis, *G. hirsutum* and *G. barbadense*. Hidden Markov Model profile of the NFYA domain (PF02045) was downloaded from the Pfam database (http://pfam.xfam.org/), and then acted as a query to screen NFYA proteins using HMMER (V3.0) software (http://hmmer.org/download.html). The conserved domain of NFYA

was verified using SMART (http://smart.embl.de) and INTERPROSCAN software (http://www.ebi.ac.uk/interpro/). Amino acid sequences of NFYA were aligned using ClustalX (version 2.0). The phylogenetic tree was generated using the maximum likelihood method under the WAG model in MEGA v5.1 (http://www.megasoftware.net/), and the reliability of interior branches was assessed with 1000 bootstrap re-samplings. Totally, 10 NFYA genes were found in Arabidopsis, 32 NFYA genes were identified in Hai7124 and TM-1. These genes can be divided into five classes. **b)** Expression patterns of NFYA family genes in responses to *Vd* infection. The expression data represent the fold change (average FPKM values of three biological replicates) compared to 0 hours. Expression patterns were visualized using MeV 4.7.0. The red box shows NFYA5 which is significantly up-regulated after *Vd* infection.

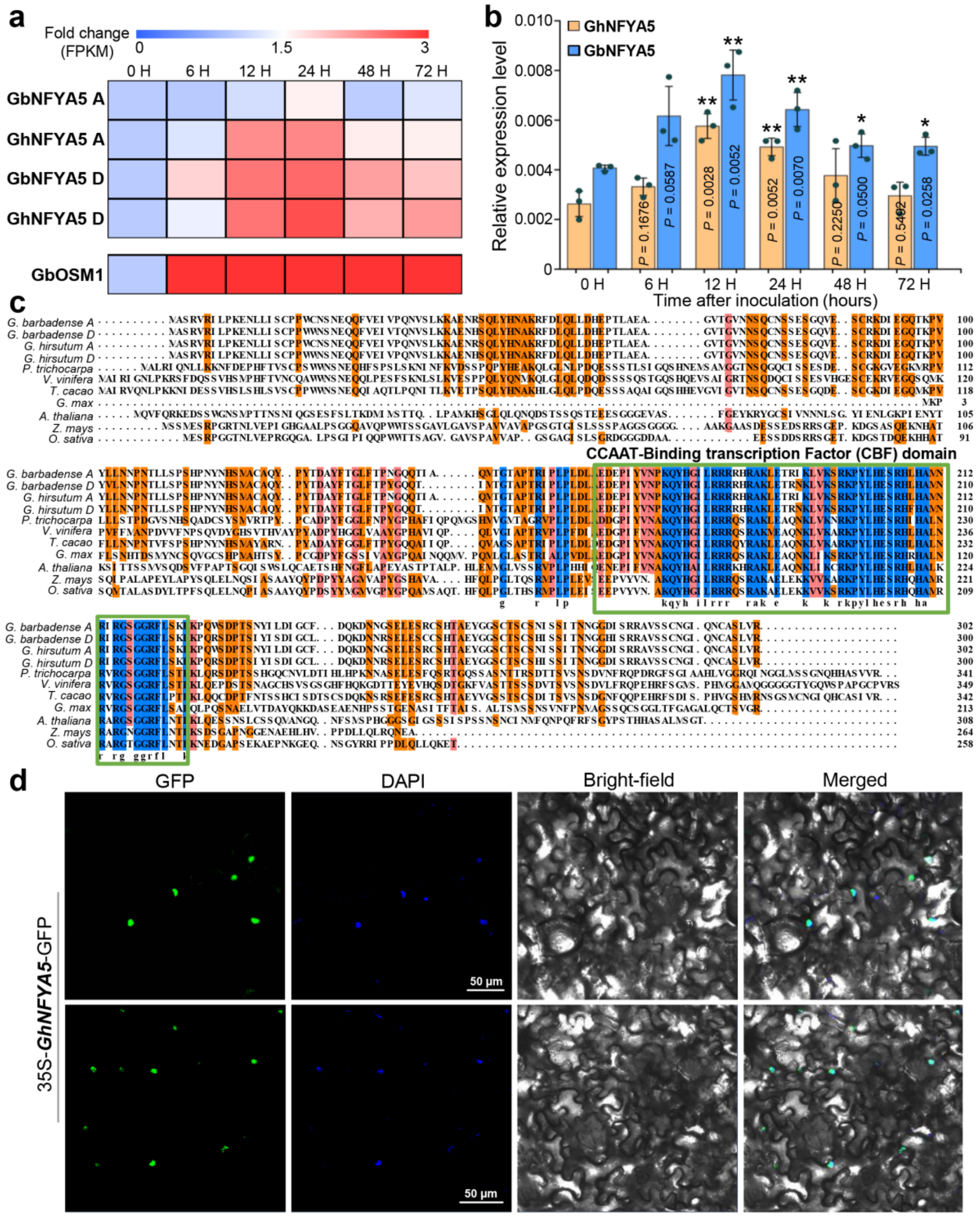


**Figure S20 Characterization of NFYA5 transcription factor in cotton. a)** Expression patterns of *NFYA5* and *GbOSM1* in response to *Vd* infection. The expression data represent the fold change (average FPKM values of three biological replicates) compared to 0 hours. Expression patterns were visualized using MeV 4.7.0. **b)** RT-qPCR analysis for *NFYA5* expression in Hai7124 and TM-1 in response to *Vd* infection. Error bars represent the standard deviation of three independent biological replicates for each experiment. The statistical analyses were performed by comparing expression level at different time points between *Vd* infection and mock treatment using Student' s *t*-test (**P*<0.05, ***P*<0.01). **c)** Amino acid sequence alignment of NFYA5 from cotton and seven

other plant species: *Populus trichocarpa*, *Vitis vinifera*, *Theobroma cacao*, *Glycine max*, *Arabidopsis thaliana*, *Zea mays*, *Oryza sativa*. The green box shows the CCAAT-Binding transcription factor domain. **d)** Localization of GhNFYA5 in tobacco epidermal cells using GFP fusion. The leaves were placed in the nucleus staining solution (1 mM DAPI) for 30 minutes before microscopic observation. The results show that GhNFYA5 is localized in the nucleus. Scale bars: 50 μm.

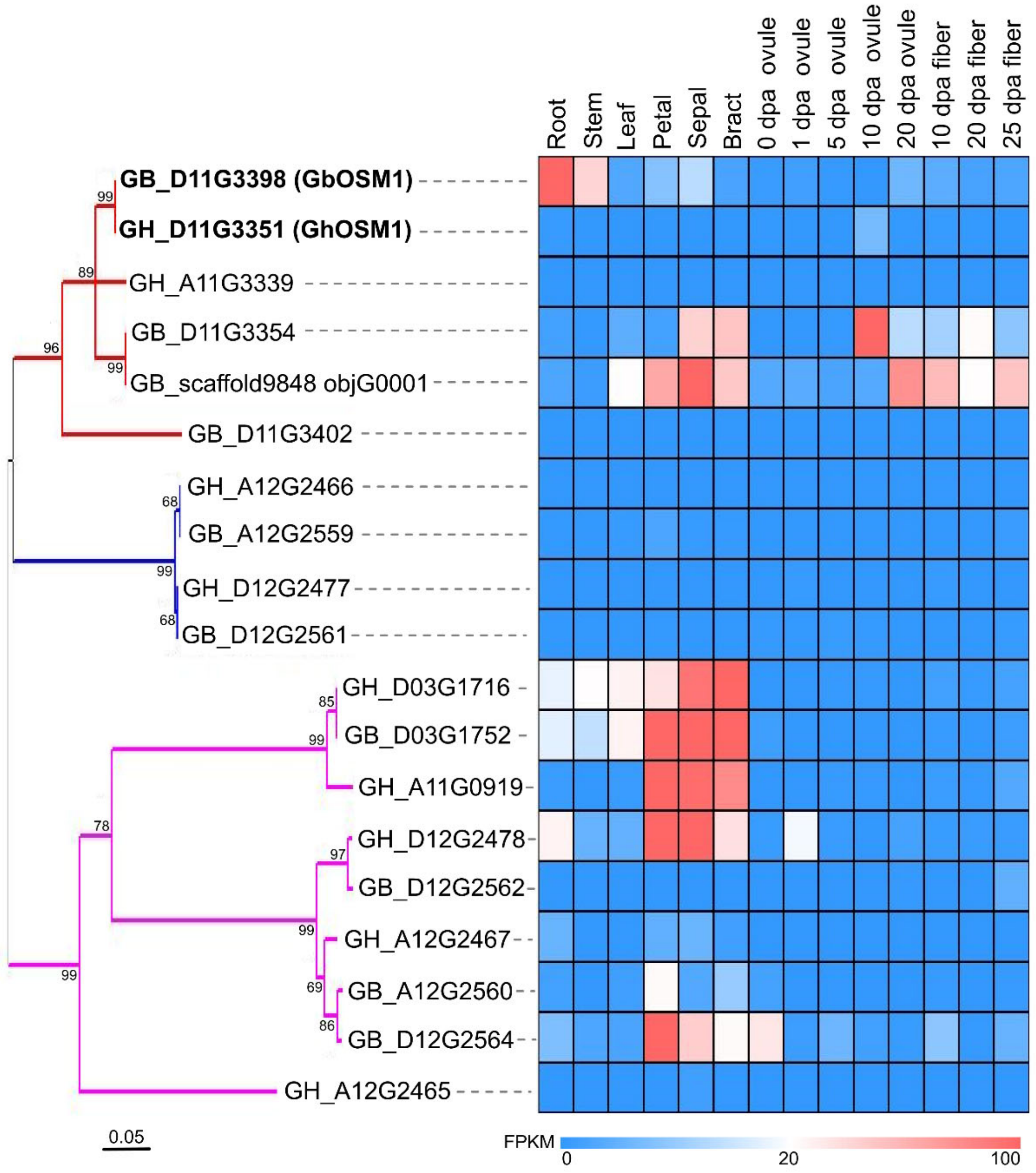


**Figure S21 Transcriptional profiling of osmotin genes in different tissues and organs in *G. hirsutum* acc. TM-1 and *G. barbadense* acc. Hai7124.** Root, stem, leaf, petal, sepal, bract, ovule at 0, 1, 5, 10 and 20 dpa, fiber at 10, 20 and 25 dpa were used for the comparative transcriptome analysis. The RNA-seq data were from http://www.ncbi.nlm.nih.gov/bioproject/ PRJNA248163/ and https://www.ncbi.nlm.nih.gov/bioproject/?term=G.+barbadense+and+Zhe jiang.

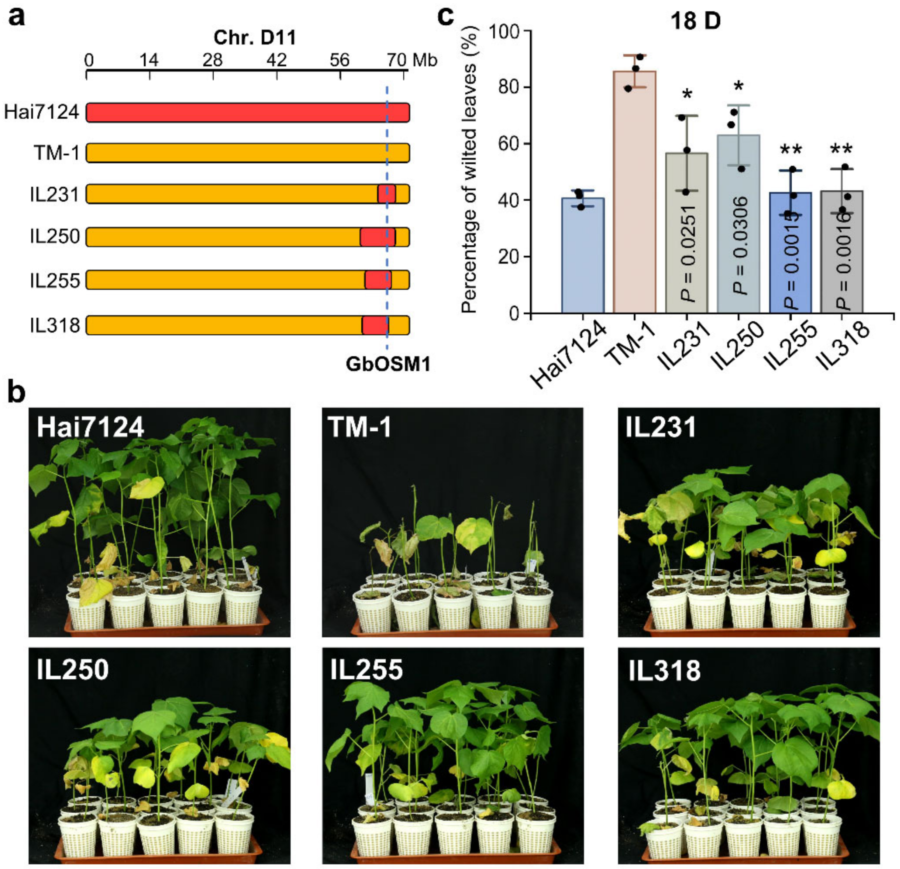


**Figure S22 Hai7124-TM-1 chromosome segment introgression lines carrying CCGAT-genotype *GbOSM1* exhibits excellent resistance to VW. a)** The schematic representation showing four Hai7124-TM-1 chromosome segment introgression lines, with Hai7124 chromosomal segments carrying *GbOSM1* in different introgression lengths. The dashed line indicates the location of the *GbOSM1* gene. **b)** Disease symptoms of four *GbOSM1* gene introgression plants at 18 days after *Vd* inoculation. **c)** Percentage of wilted leaves in four *GbOSM1* gene introgression plants after *Vd* inoculation. Each biological replicate contains at least 30 seedlings. Error bars represent the standard deviation of three biological replicates. The statistical analyses were performed by comparing the percentage of wilted leaves of introgression plants with TM-1 using Student' s *t*-test (**P*<0.05, ***P*<0.01).

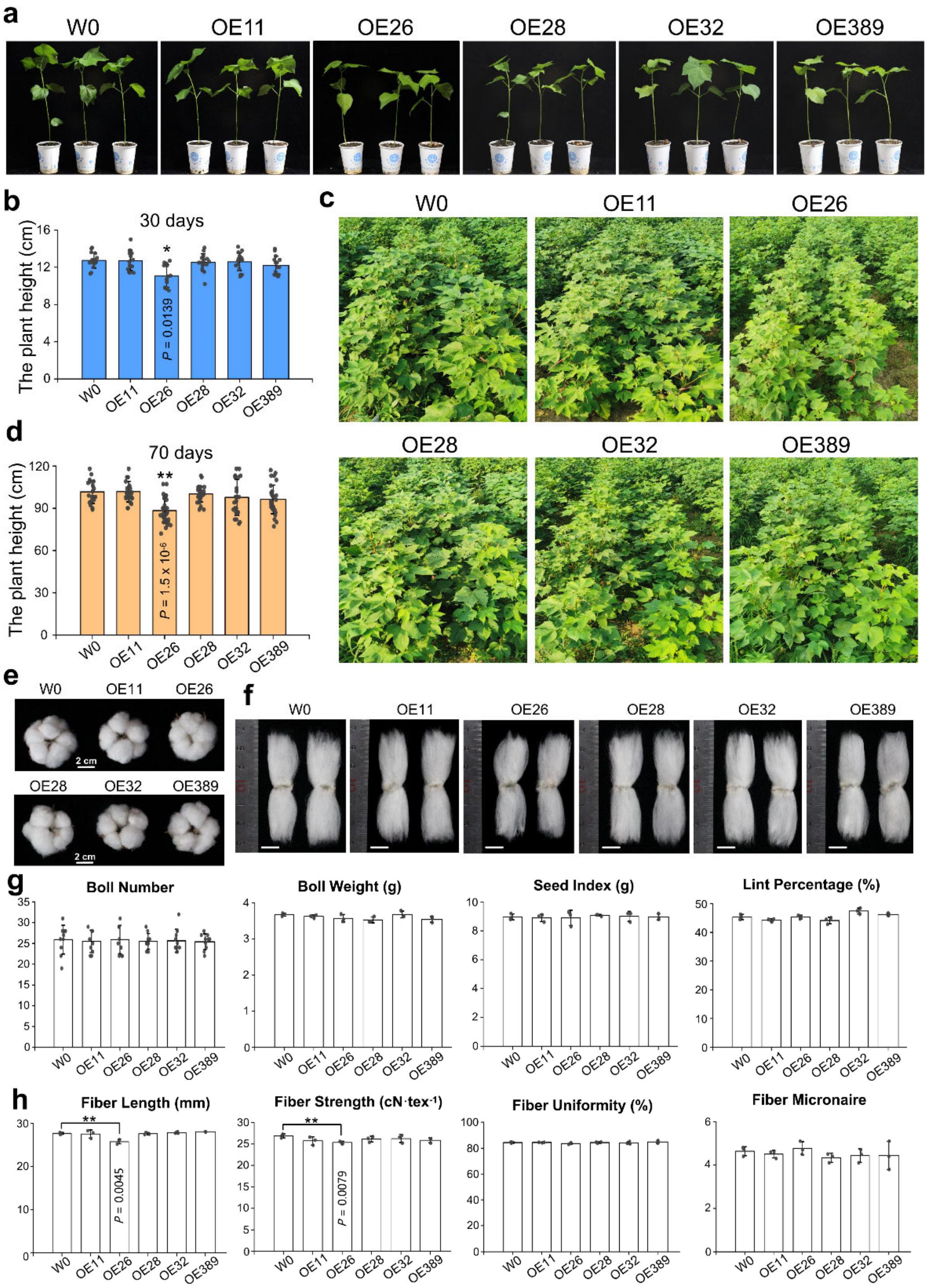


**Figure S23 Investigation of plant height, yield components and fiber quality traits in overexpressing *GbOSM1* transgenic lines. a-b)** Phenotype of four *GbOSM1*-overexpressing cotton lines and control plants were observed at 30 days, under a 16-hours light/8-hour dark cycle at 28/25 °C conditions. The stem height above the cotyledons was measured in the different transgenic cotton seedlings. n > 15, statistical analyses were performed using Student's *t*-test

(**P*<0.05). **c-d)** Plant height of *GbOSM1*-overexpressing transgenic cotton lines grown in the field for 70 days. n > 20, statistical analyses were performed using Student's *t*-test (***P*<0.01). **e)** Phenotypic analysis of mature cotton bolls in WT and *GbOSM1* transgenic plants. Scale bars: 2 cm. **f)** Phenotypic analysis of mature fibers in WT and *GbOSM1* transgenic plants. Scale bars: 1 cm. **g-h)** Comparison of yield components and fiber quality traits between *GbOSM1* transgenic lines and wildtype. Asterisks indicate statistically significant differences, as determined by the Student's *t*-test (***P*<0.01).

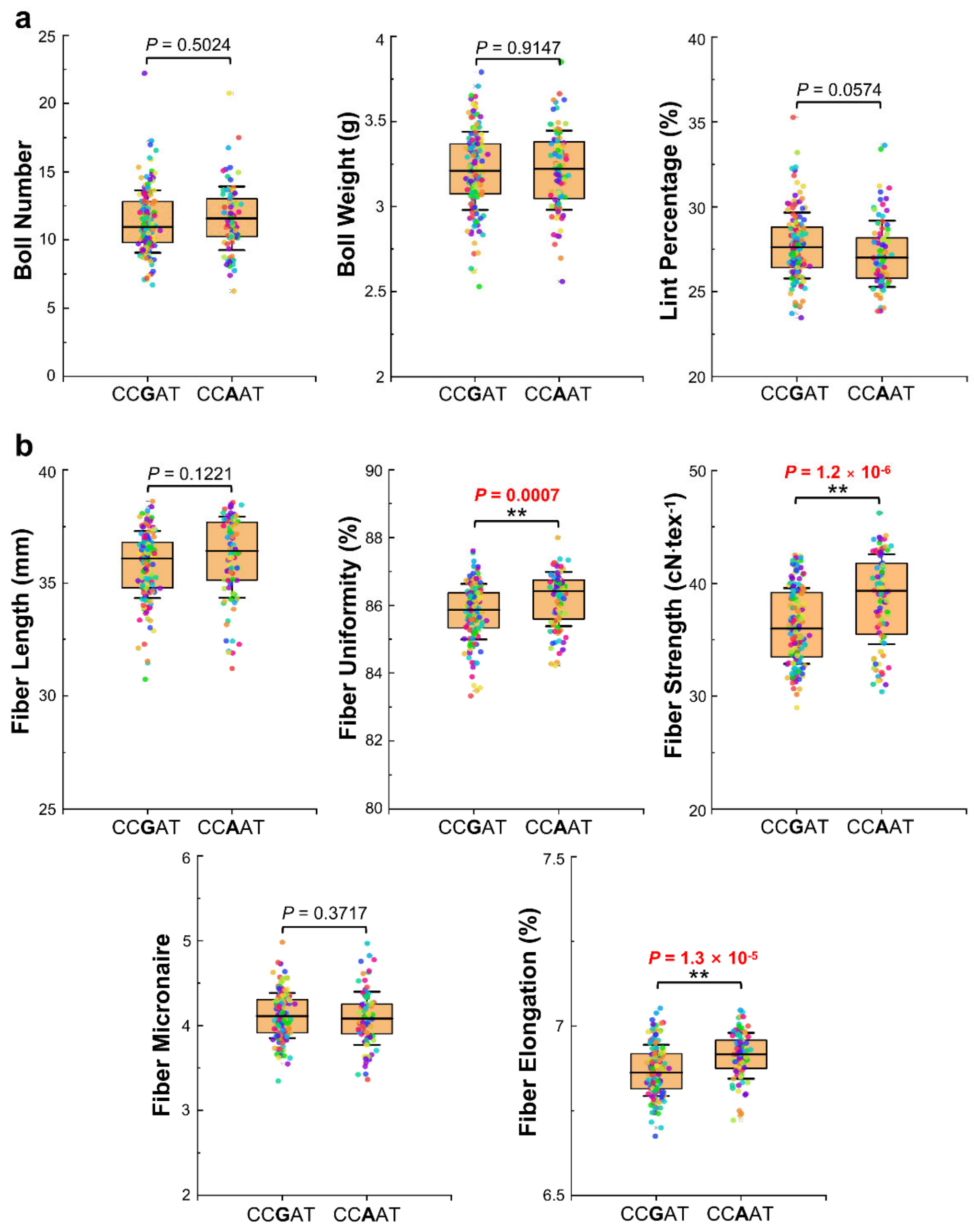


**Figure S24 Statistical analysis of yield components and fiber quality traits between the CCAAT and CCGAT haplotypes in 269 *G. barbadense* accessions. a)** Comparison of boll number, boll weight and lint percentage traits between *G. barbadense* accessions carrying CCAAT/CCGAT haplotypes. **b)** Comparison of fiber length, fiber uniformity, fiber strength, fiber micronaire and fiber elongation between *G. barbadense* accessions carrying CCAAT/CCGAT haplotypes. In box plots, different colors represent different *G. barbadense* accessions, center line indicates median, box limits denote upper and lower quartiles. Student's *t*-test demonstrates

significant differences (***P*<0.01) between the two haplotypes. CCAAT haplotype, n=106; CCGAT haplotype, n=163.
